# Current progress, challenges and future prospects of indazoles as protein kinase inhibitors for the treatment of cancer

**DOI:** 10.1039/d1ra03979b

**Published:** 2021-07-20

**Authors:** Nitin Tandon, Vijay Luxami, Divya Kant, Runjhun Tandon, Kamaldeep Paul

**Affiliations:** School of Chemical Engineering and Physical Sciences, Lovely Professional University Phagwara-144411 India gupta.runjhun@gmail.com; School of Chemistry and Biochemistry, Thapar Institute of Engineering and Technology Patiala-147004 India kpaul@thapar.edu

## Abstract

The indazole core is an interesting pharmacophore due to its applications in medicinal chemistry. In the past few years, this moiety has been used for the synthesis of kinase inhibitors. Many researchers have demonstrated the use of indazole derivatives as specific kinase inhibitors, including tyrosine kinase and serine/threonine kinases. A number of anticancer drugs with an indazole core are commercially available, *e.g.* axitinib, linifanib, niraparib, and pazopanib. Indazole derivatives are applied for the targeted treatment of lung, breast, colon, and prostate cancers. In this review, we compile the current development of indazole derivatives as kinase inhibitors and their application as anticancer agents in the past five years.

## Abstract 2

The indazole core is an interesting pharmacophore due to its applications in medicinal chemistry.

## Graphical abstract

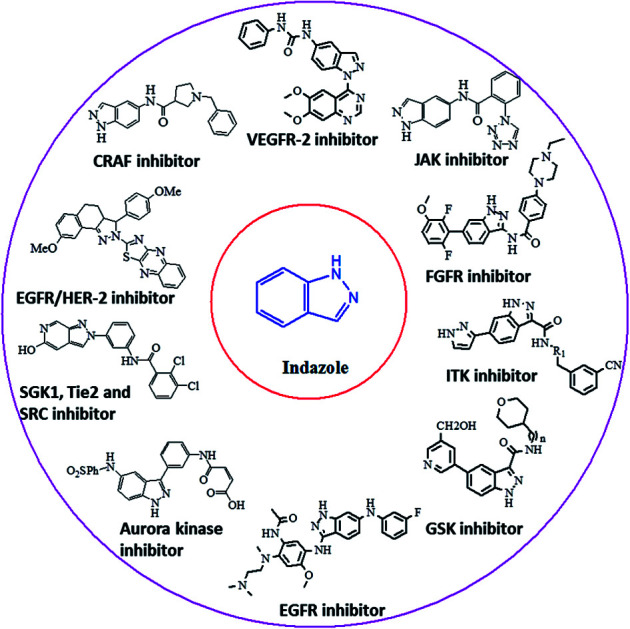

## 1. Introduction

Cancer is a disease that causes uncontrollable abnormal cell growth, which can start in any organ of the body and further spread to distant organs. Cancer is the second leading cause of deaths globally, which has led to almost 9.6 million deaths in 2018 and accounted for 1 out of 6 deaths according to the World Health Organization (WHO).^1^ The treatment of cancer includes surgery, radiation therapy, chemotherapy, immunotherapy, hormonal therapy, targeted therapy and synthetic lethality. The location of cancer cells, the stage of cancer, the age of the patient and the general state of the patient are some of the factors that decide the therapy to be used. The probability of cancer cells affecting the adjacent tissues or organs limits the use of surgery, whereas the use of chemotherapy and radiation therapy is associated with negative effects on normal cells.^2–5^ One of the possible pathways leading to this deadly disease is the mutation of various types of genes including kinases, which can trigger diverse cellular anomalies, leading to the initiation of cancer. The human genome includes 518 protein kinases, where the most probable mutations occur in BRAF,^6,7^ KIT,^8^ EGFR^9^ and FTL3,^10^ whereas some kinases are regulated by epigenetic mechanisms such as RET,^11,12^ AATK,^13^ EPHA5,^14^ CHK2 (ref. 15) and PKD1.^16^ Phosphorylation is one of the important steps involved in the cell cycle, growth, apoptosis, motility, proliferation, *etc.* Inhibition of kinase activity has a profound effect on this process. In addition, mutation or de-regularization of kinase activity has been proven to be oncogenic and has potential to inhibit the spread of cancer cells.^17^ Considering that kinases play an important role in cell biology, many attempts have been made to develop novel kinase inhibitors such as tyrosine kinase,^18^ cyclin-dependent kinase,^19–21^ aurora kinase,^22,23^ EGFR^24^ and VEGFR,^25^ which can serve as potential drugs candidates.

**Fig. 1:**
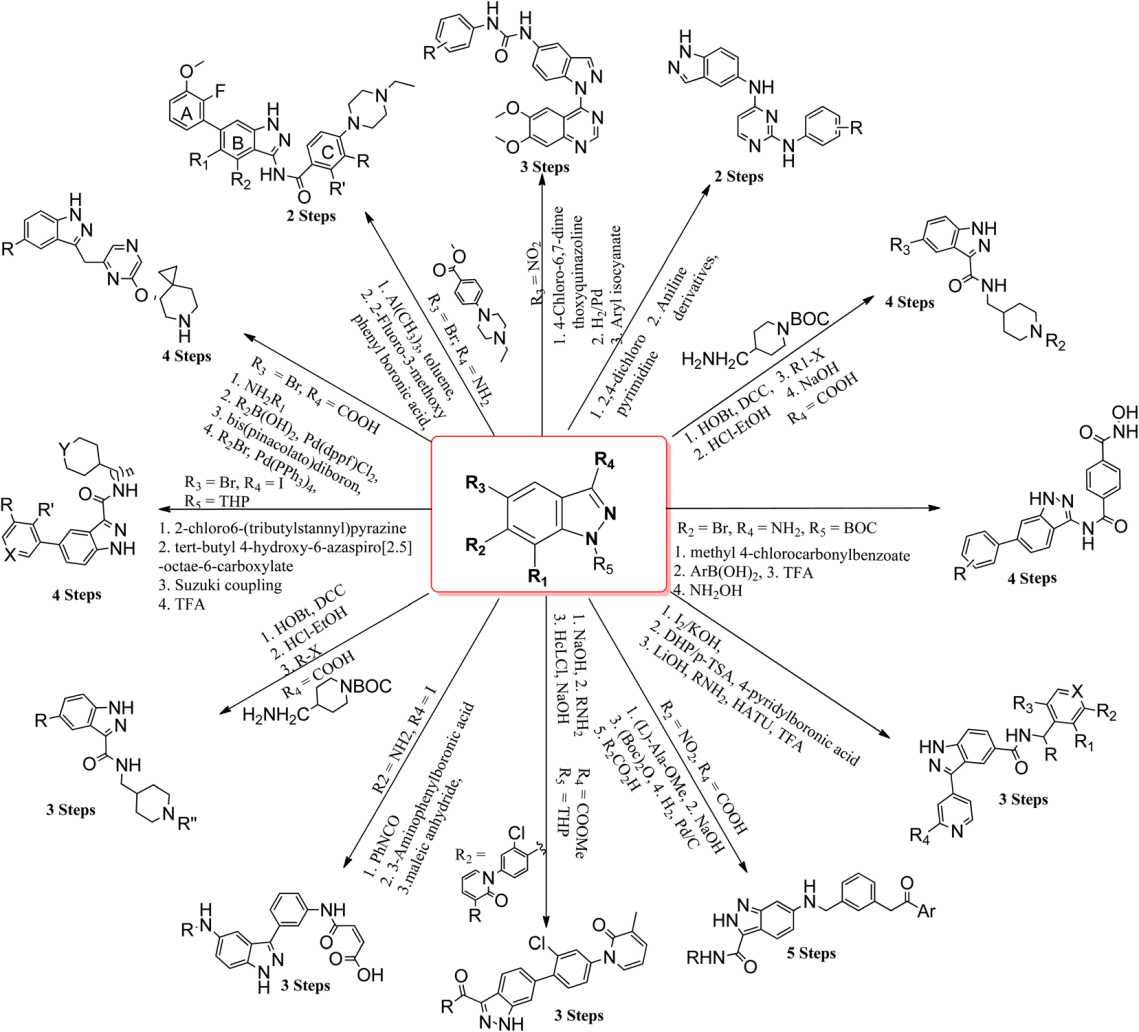
Pathways for the synthesis of indazole derivatives.

Many derivatives of primidine,^26^ benzimidazole,^27^ benzothiazole,^28^ coumarin,^29^ naphthalimide,^30,31^*etc.* have been used as potential kinase inhibitors as possible treatment for cancer. Indazole derivatives possess a wide range of pharmacological activities such as antibacterial, antifungal, anti-inflammatory, anti-HIV, anti-arrhythmic and anti-tumor.^32–37^ The aim of this review is to compile the work performed by different research groups in the field of indazole derivatives as kinase inhibitors. These indazole derivatives have been synthesized using various synthetic methodologies (Fig. 1). Further, the data has been compiled from 2015–2021, which will be beneficial to researchers for the design and synthesis of novel indazole derivatives with desired therapeutic outcomes.

## 2. Mono-kinase inhibitors

### (i) Vascular endothelial growth factor receptor 2 (VEGFR-2) inhibitors

Vascular endothelial growth factor receptor is a signaling protein involved in angiogenesis and vasculogenesis. There are three types of VEGFR, namely VEGFR-1, VEGFR-2 and VEGFR-3. VEGFR-2 is known to mediate all cellular responses of VEGFR.^38^ They are also known as kinase insert domain receptor (KDR) inhibitors, which can affect multiple signaling pathways such as tumor angiogenesis, proliferation and metastasis. VEGFR has been found to be overexpressed in many cancer types such as melanoma, medulloblastoma and ovarian cancer.^39^ A reduction in VEGFR-2 results in a reduction in blood flow to tumor cells, thereby suppressing tumor angiogenesis.^40^

**Fig. 2:**
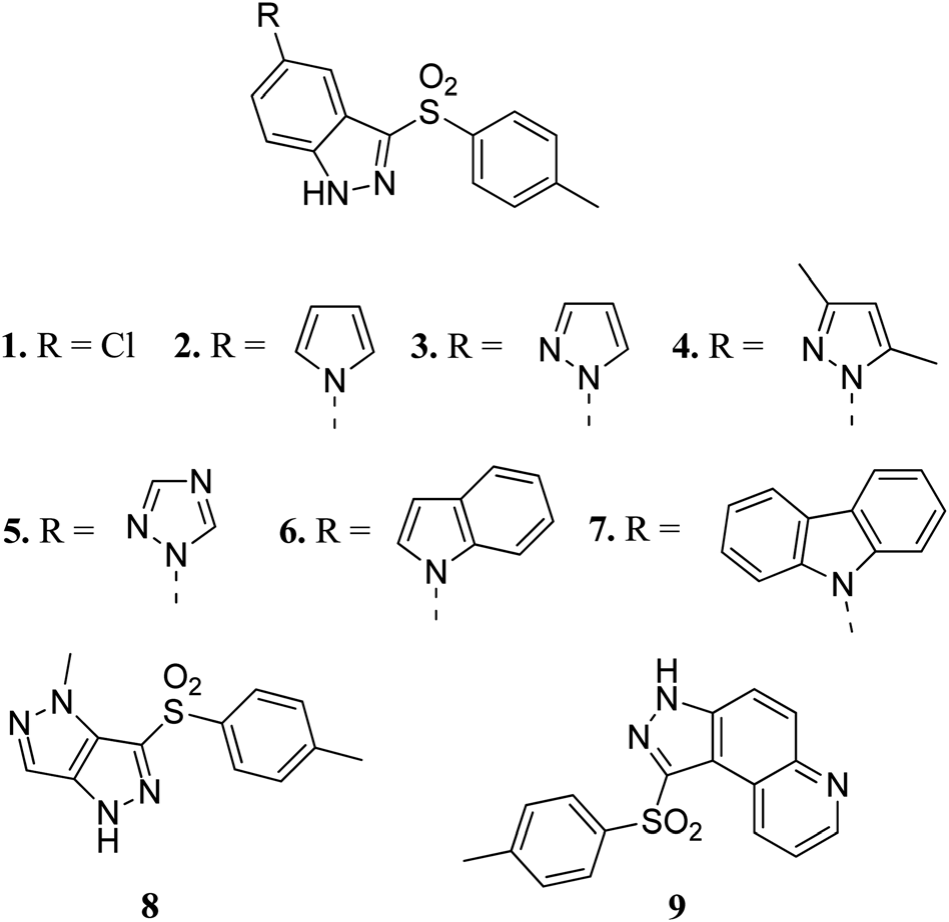
Arylsulphonyl indazoles as VEGFR2 kinase inhibitors.

Czaja *et al.* studied arylsulphonyl indazole derivatives (Fig. 2) and their interactions with VEGFR2 kinase using docking, MD simulation and other computational methods.^41^ These studies suggested that compounds 1–6 and 9 are superimposable in their docked conformers with protein (PDB code: 3EWH)^42^ having an interaction energy in the range of −36.5 to −66.5 kcal mol^−1^. However, the dissimilar orientation of the indazole ring in compound 7 led to different sets of interactions with VEGFR2 kinase compared to the other derivatives. Compounds 1–6 and 9 were stabilized by hydrogen bonding and π–π stacking. Further exploration of these compounds for their cytotoxic activity against the human colon adenocarcinoma cancer cell line (HT-29), Michigan Cancer Foundation-7 cancer cell line (MCF-7) and MD Anderson-Metastatic Breast-231 cancer cell line (MDA-MB-231) suggested that compounds having pyrazole (3), indole (6) and carbazole (7) moieties possessed good cytotoxic activity, whereas compounds having chloro (1) and pyrazole (8) moieties were observed to be the weakest cytotoxic agents.

**Fig. 3:**
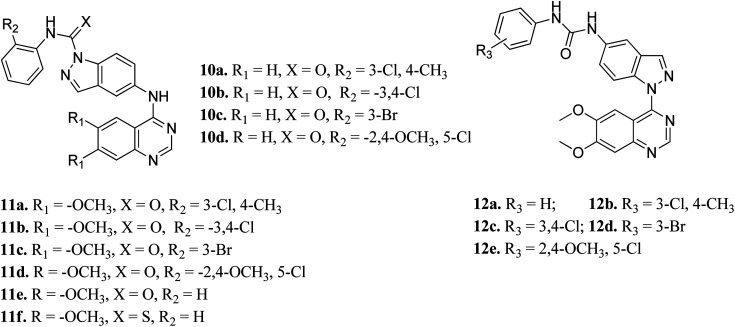
Indazole derivatives with different substitution patterns as VEGFR-2 kinase inhibitors.

Elsayed *et al.* reported the activity of a series of quinazoline derivatives of indazole (Fig. 3) against VEGFR-2.^43^ Derivatives 11a–f exhibited better activity than 10a–d because of the presence of methoxy group(s) on the benzamide ring, with 11a being the most potent derivative, whereas thio derivative 11f exhibited the lowest activity. Interestingly, 12a–e possessed excellent activity against VEGFR-2, with 12b, 12c and 12e exhibiting better activity (IC_50_ values of 5.4, 5.6 and 7 nM, respectively) than sorafenib (IC_50_ = 90 nM). An increase in bulkiness at the terminal phenyl ring resulted in better activity in the case of 12b, 12c and 12e. Further, compound 11a exhibited significant inhibitory activity against a non-small cell lung cancer cell line (NCI-H322M) (GI = 60%) and human breast adenocarcinoma cancer cell line (MDAMB-468) (GI = 90%), whereas 12b was found to be selective against a colorectal cancer cell line (KM12) (GI = 74%). Derivatives 12a and 12c showed profound anticancer activity against a full panel cell line with 12a showing sub-micromolar activity.

**Fig. 4:**
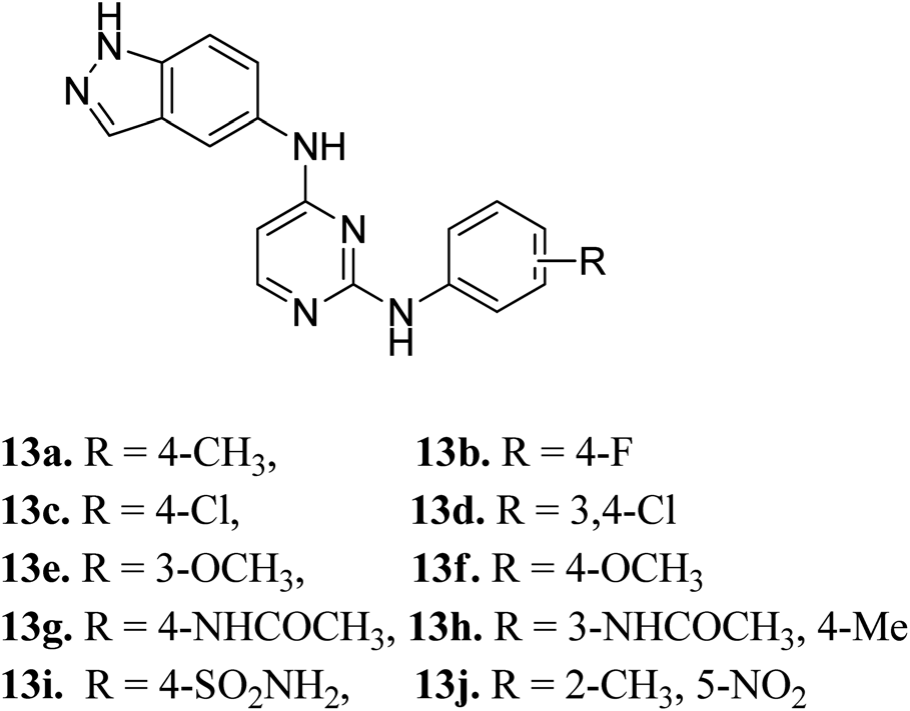
Indazole–pyrimidine-based derivatives as VEGFR-2 inhibitors.

Later, Elsayed *et al.* reported indazole–pyrimidine-based derivatives substituted at the 2-position of the pyrimidine ring as VEGFR-2 inhibitors, where compound 13a–j exhibited better inhibitory properties (Fig. 4).^44^ The presence of hydrophobic groups such as alkyl or halogen (13a–d) led to a decrease in potency compared to a methoxy derivative (13e and f). Conversely, hydrogen bond-forming groups such as amide (13f and 13g with IC_50_ = 114 and 57.9 nM, respectively) and sulfonamide (13i, IC_50_ = 34.5 nM) resulted in enhanced activity compared to pazopanib (IC_50_ = 30 nM).

### (ii) Fibroblast growth factor receptor (FGFR) inhibitors

Fibroblast growth factor receptor (FGFR) inhibitors are a class of tyrosine kinase receptors, which play an important role in cell development and growth.^45,46^ They can be categorized as FGFR1 to FGFR4 with a variation in their sequence identity from 56–71%.^47^ FGFRs play a crucial role in important physiological processes such as cell migration, survival, proliferation and differentiation.^48–50^ Dysregulation of FGFR is associated with many types of cancers such as ovarian cancer, urothelial carcinoma, lung adenocarcinoma and hepatocellular carcinoma.^51,52^ Erdafitinib, a pan-FGFR inhibitor, has been approved by the USFDA for the treatment of urothelial carcinoma.

**Fig. 5:**
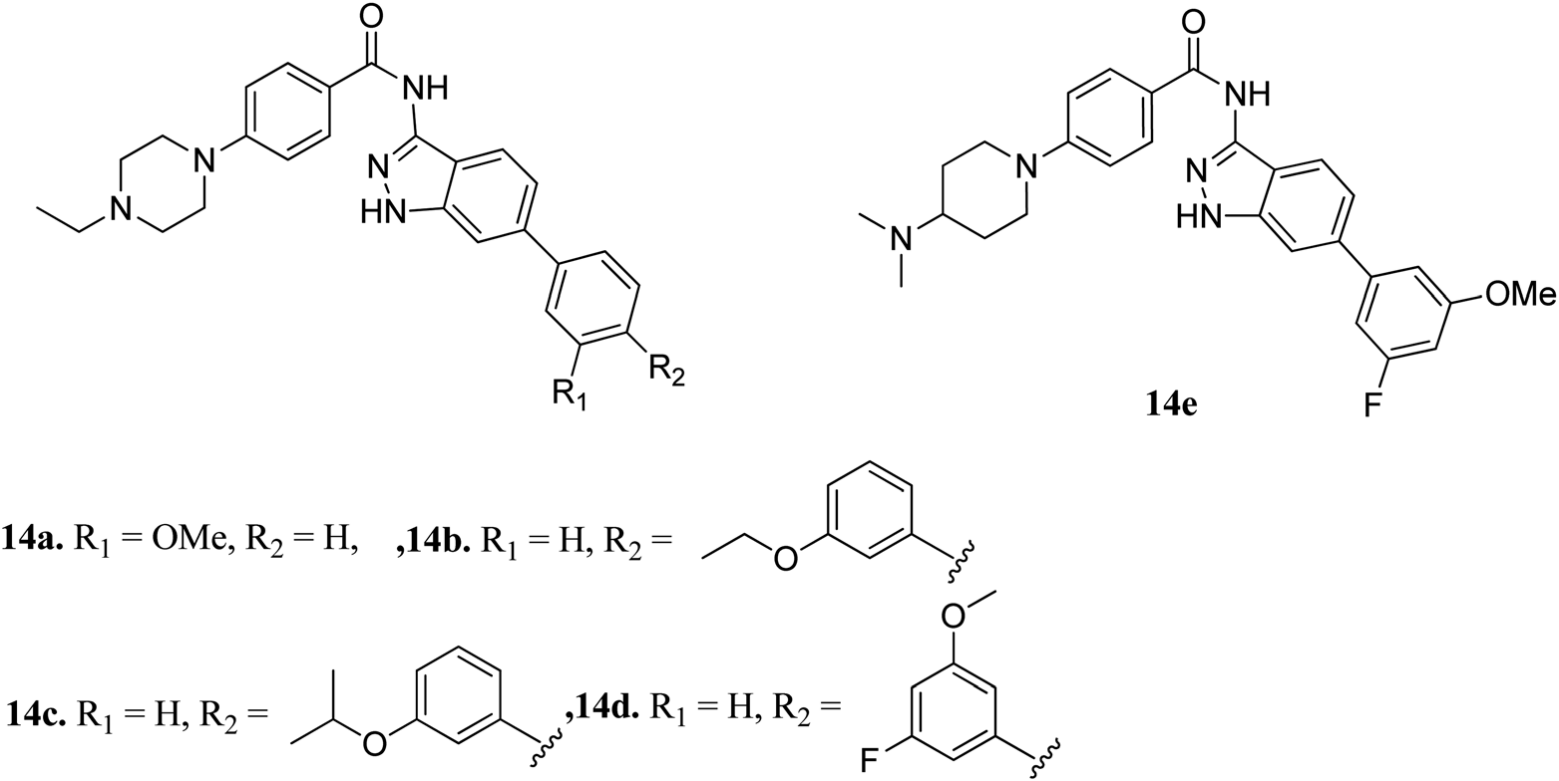
Indazole derivatives as inhibitors of FGFR1.

Liu *et al.* reported derivatives with varying substitution at the phenyl ring of indazole as FGFR1 inhibitors (Fig. 5).^53^ The structure–activity relationship (SAR) studies of the synthesized derivatives (14a–e) revealed that the substitution of the 3-methoxyphenyl group on the phenyl ring (14a, IC_50_ = 15 nM) with larger groups such as 3-ethoxyphenyl (14b, IC_50_ = 13.2 nM) and 3-isopropoxyphenyl (14c, IC_50_ = 9.8 nM) led to an increase in activity. Interestingly, the presence of an additional fluorine atom on the phenyl ring led to a remarkable improvement in activity (14d, IC_50_ = 5.5 nM) compared to its counterpart 14a (IC_50_ = 15 nM). Moreover, 14e was found to be selective against FGFR1 compared to the other FGFR, anaplastic lymphoma kinase (ALK), ephrin type-A receptor 2 (EPH-A2) and breakpoint cluster region protein (Bcr-Abl). Further, docking studies of these compounds in the ATP binding pocket of FGFR1 predicted various interactions such as hydrogen bonding and hydrophobic interactions through the indazole N–H and 3-methoxyphenyl group.

**Fig. 6:**
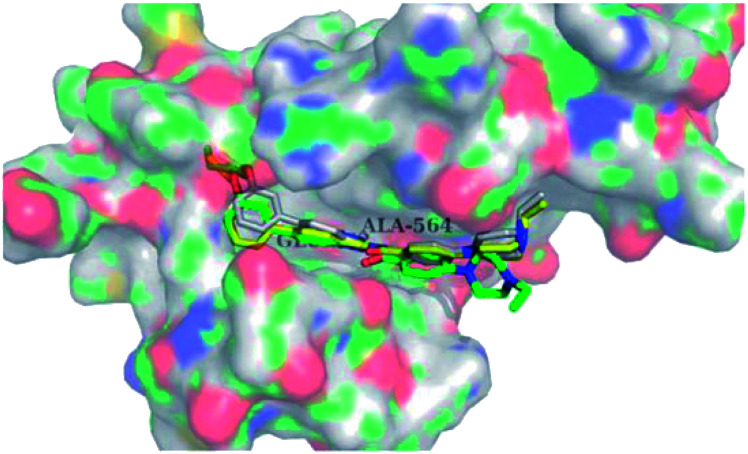
Superimposed docking structures of 14b (white) and 14c (yellow) with FGFR1 (4ZSA) (no permission required).

Docking studies of 14b and 14c with the ATP-binding pocket of FGFR1 (4ZSA) revealed that the N–H of the indazole ring formed a hydrogen bond with Glu562, whereas the nitrogen atom of the indazole group and N–H of the amide bond formed a hydrogen bond with Ala564. The corresponding ethoxy and iso-propoxy groups of compounds 14b and 14c filled the hydrophobic cavity tightly, resulting in the high potency of these derivatives (Fig. 6).

**Fig. 7:**
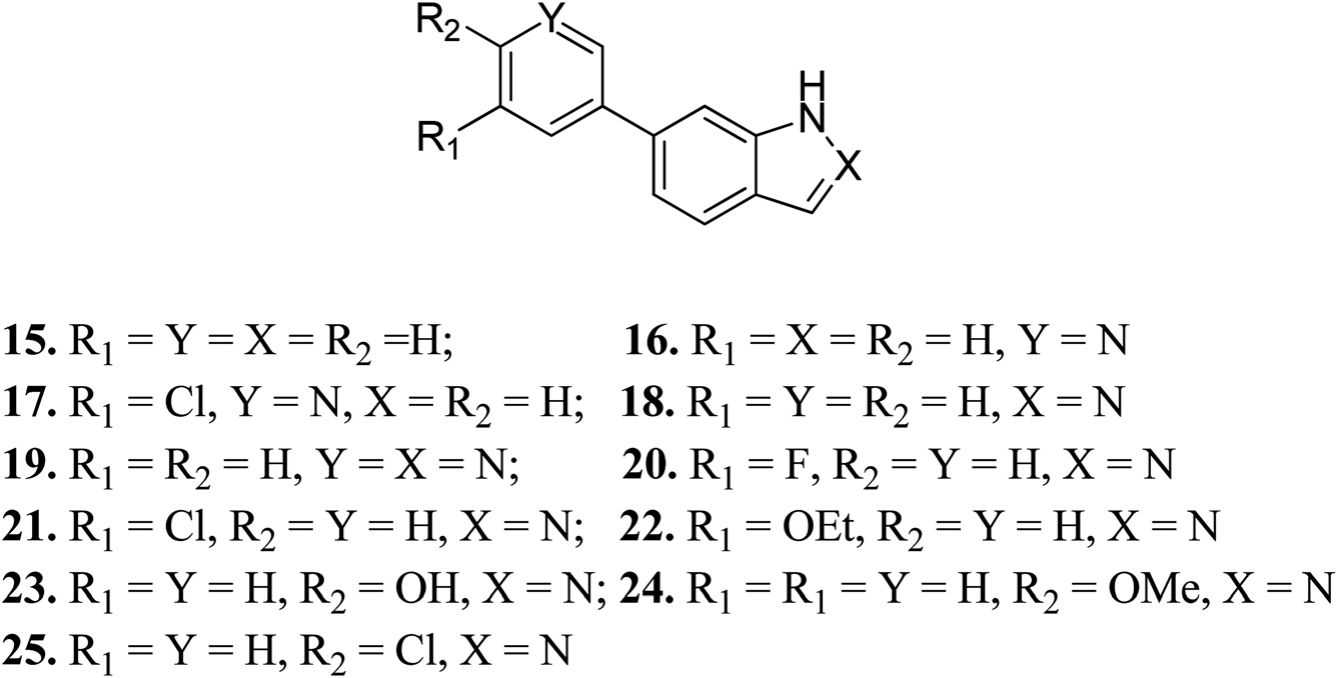
Structure-based design of indazole derivatives as inhibitors of FGFR1 kinases.

Turner *et al.* reported the structure-based design and synthesis of indazole derivatives (Fig. 7) for their exploration as inhibitors of FGFR1 kinases.^54^ The initial screening supported the importance of the indazole group with nitrogen at the 2-position, given that compounds 15–17 did not show the required inhibition against FGFR-1. Further, the presence of a pyridine ring led to a decrease in IC_50_ value for compound 19 (IC_50_ = 90 μM) compared to compound 18 (IC_50_ = 77 μM) bearing a phenyl substituent. Compound 22 was also found to inhibit FGFR-2 and FGFR-3 with IC_50_ values of 0.8 and 4.5 μM, respectively, and possessed 2.5-fold activity against FGFR-2 over FGFR-1. Compound 23 exhibited an IC_50_ value of 2 μM compared to 24 and 25, emphasizing the importance of the H-donor OH group.

**Fig. 8:**
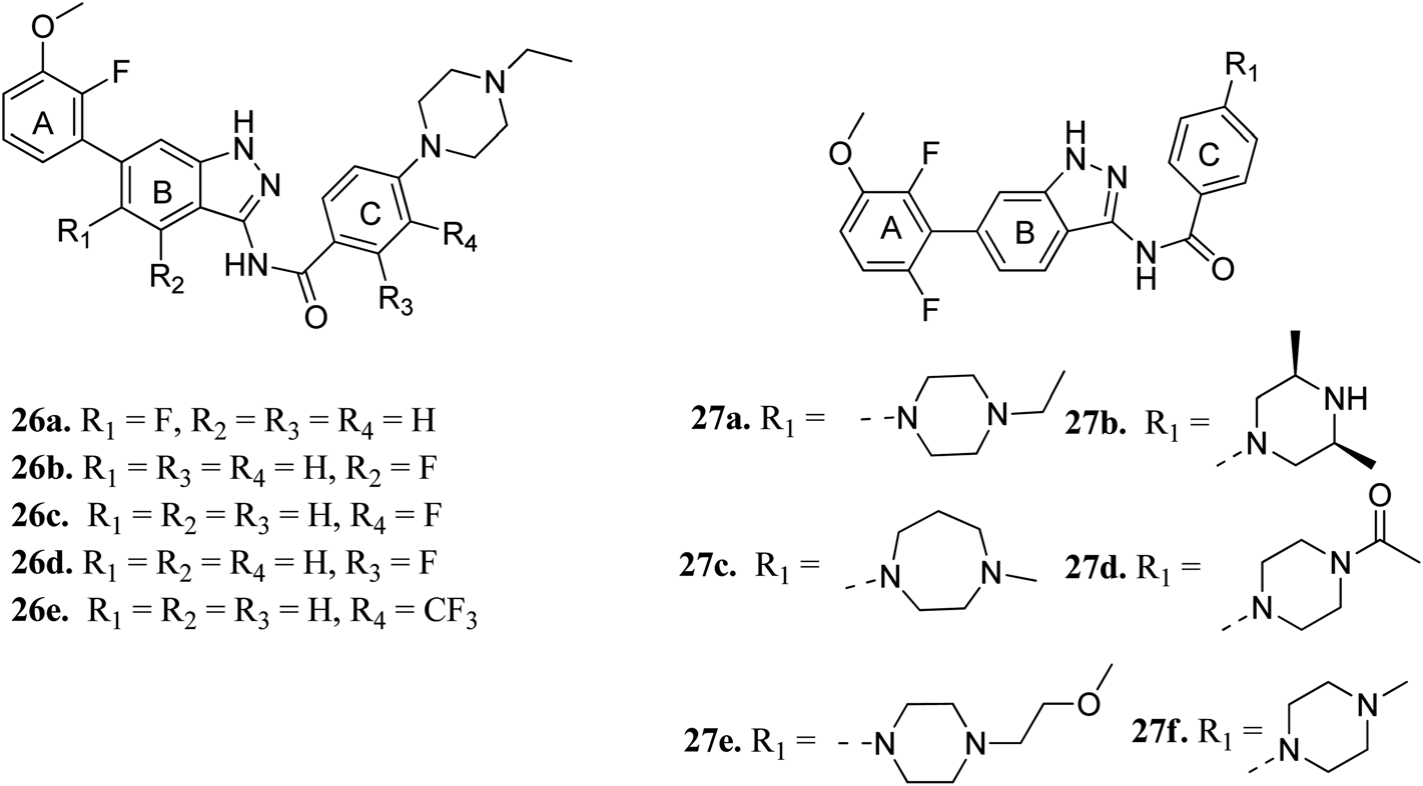
1*H*-Indazol-3-amine derivatives as inhibitors of fibroblast growth factor receptor.

Cui *et al.* reported 1*H*-indazol-3-amine derivatives (Fig. 8) as inhibitors of fibroblast growth factor receptors FGFR1 and FGFR2.^55^ Their studies suggested that the presence of fluorine substitution in ring B or ring C was not tolerated (26a–e). However, the presence of fluorine substitution at 6-position of ring A resulted in improved enzymatic activity and cellular potency (27a, FGFR1 IC_50_ < 4.1 nM, FGFR2 IC_50_ = 2.0 nM, Koeffler Golde-1 (KG1) IC_50_ = 25.3 nM, and (SNU) IC_50_ = 77.4 nM). Further, compounds 27a, 27c and 27f were found to exhibit better activity than 27b, 27d and 27e. In addition, compound 27a showed high affinity towards the FGFR1 enzyme (PDB code: 4ZSA^56^) in the docking studies.

**Fig. 9:**
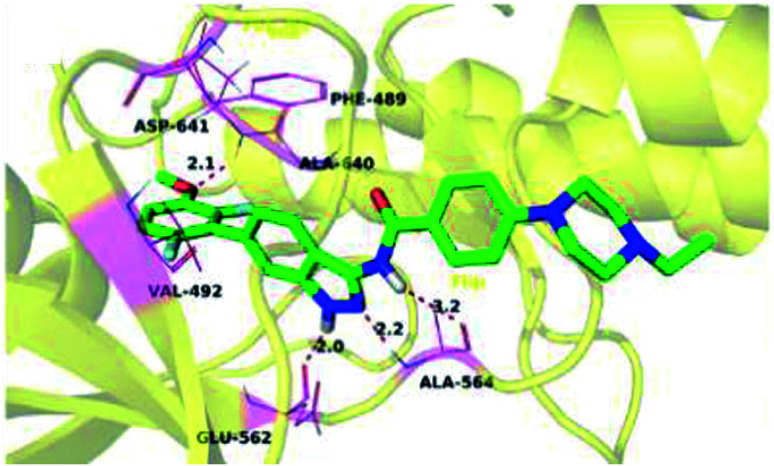
Predicted docking of 27a with FGFR1 (reproduced with permission; License Number: 5087581066045).

The binding mode of 27a with FGFR1 (Fig. 9) revealed that this derivative was tightly bound to the ATP binding site of FGFR1. The 3-aminoindazole group occupied the hinge region and formed three hydrogen bonds with Ala564 and Glu562, whereas the phenyl ring of the indazole moiety participated in π–π stacking with Phe489. Interestingly, the methoxy oxygen of 27a participated in hydrogen bond formation with Asp641, whereas the fluorine atoms formed hydrophobic interactions with Val492 and Ala640.

**Fig. 10:**
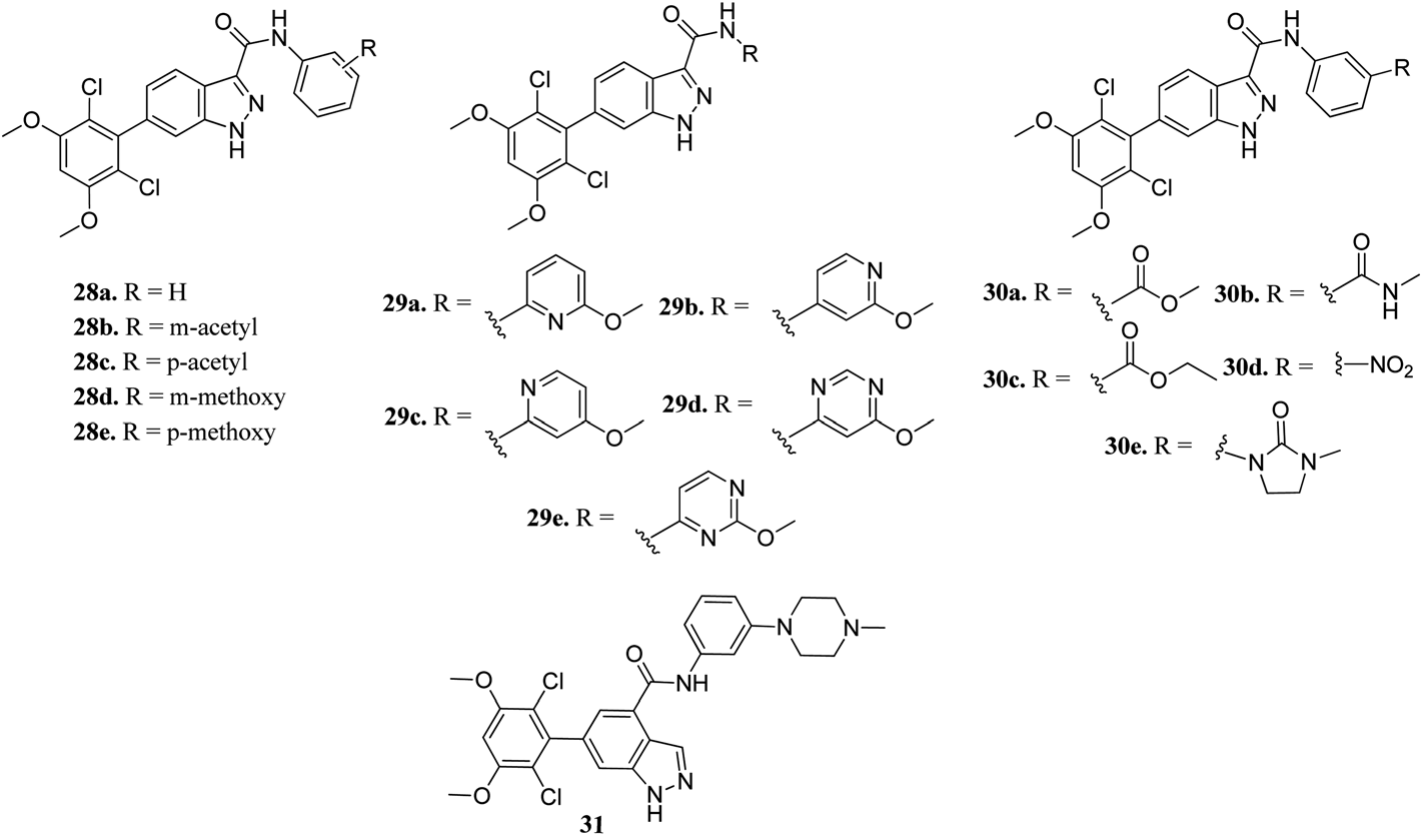
6-(2,6-Dichloro-3,5-dimethoxyphenyl)-4-substituted-1*H*-indazoles as FGFR inhibitors.

Zhang *et al.* reported 6-(2,6-dichloro-3,5-dimethoxyphenyl)-4-substituted-1*H*-indazole derivatives (Fig. 10) as FGFR inhibitors.^57^ The introduction of an acetyl or methoxy group at the *meta* position of the phenyl ring of 28a (enzyme inhibition at 58.8 μM/with IC_50_ = 69.1 nM) led to an increase in activity against FGFR1 (enzyme inhibition: 76.4 μM L^−1^ and 57.0 μM L^−1^ for 28b and 28d, respectively) compared to the *para* position (enzyme inhibition: 32.2 μM L^−1^ and 11.9 μM L^−1^ for 28c and 28e, respectively). Substitution by a methylcarbamoyl group enhanced the activity (30b, IC_50_ = 38.6 nM) and the 4-methylpiperazine analogue (31) exhibited remarkable activity (IC_50_ = 30.2 nM).

**Fig. 11:**
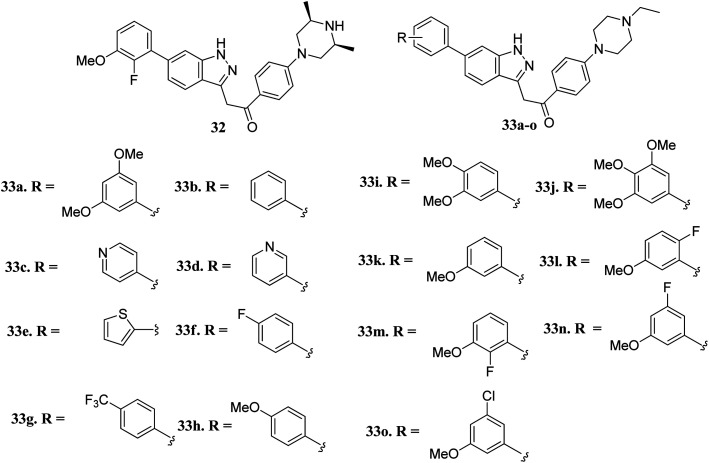
Novel indazole derivatives as inhibitors of FGFR.

Liu *et al.* reported novel methylketo derivatives containing a piperazine moiety (Fig. 11) as inhibitors of FGFR.^58^ Modification at the piperidine ring of 32 led to a decrease in the activity of 33a against FGFR1 from an IC_50_ value of 14.6 to 25 nM. However, the replacement of the phenyl ring of 33b (IC_50_ = 40.4 nM) with pyridine (33c and d, IC_50_ > 1000 nM), thiophene (33e, IC_50_ > 1000 nM) and introduction of a fluorophenyl (33f), trifluoromethyl phenyl (33g) or methoxyphenyl (33h–j) group at the *para* position led to a decrease in activity with IC_50_ values near 1000 nM. Interestingly substitution with a methoxy group at the *meta* position of the phenyl ring led to improved activity (33k, FGFR1 IC_50_ = 15 nM; Seoul National University cancer cell line (SNU-16) IC_50_ = 642.1 nM). Further optimization of 33b with a halogen moiety at the *ortho* or *meta* position of the phenyl ring led to enhanced activity (33m–o, FGFR1 IC_50_ = 2.9, 5.5 and 9.6 nM, respectively).

### (iii) Epidermal growth factor receptor (EGFR) inhibitors

Epidermal growth factor receptor (EGFR) kinases also belong to the family of tyrosine kinases. Overexpression of EFGR is associated with gene mutation and amplification, resulting in cell survival, invasion, metastasis, proliferation and prognosis. These kinases have been approved for the treatment of pancreatic, lung, and colorectal cancer and squamous cell carcinoma of the neck and head.^59^

**Fig. 12:**
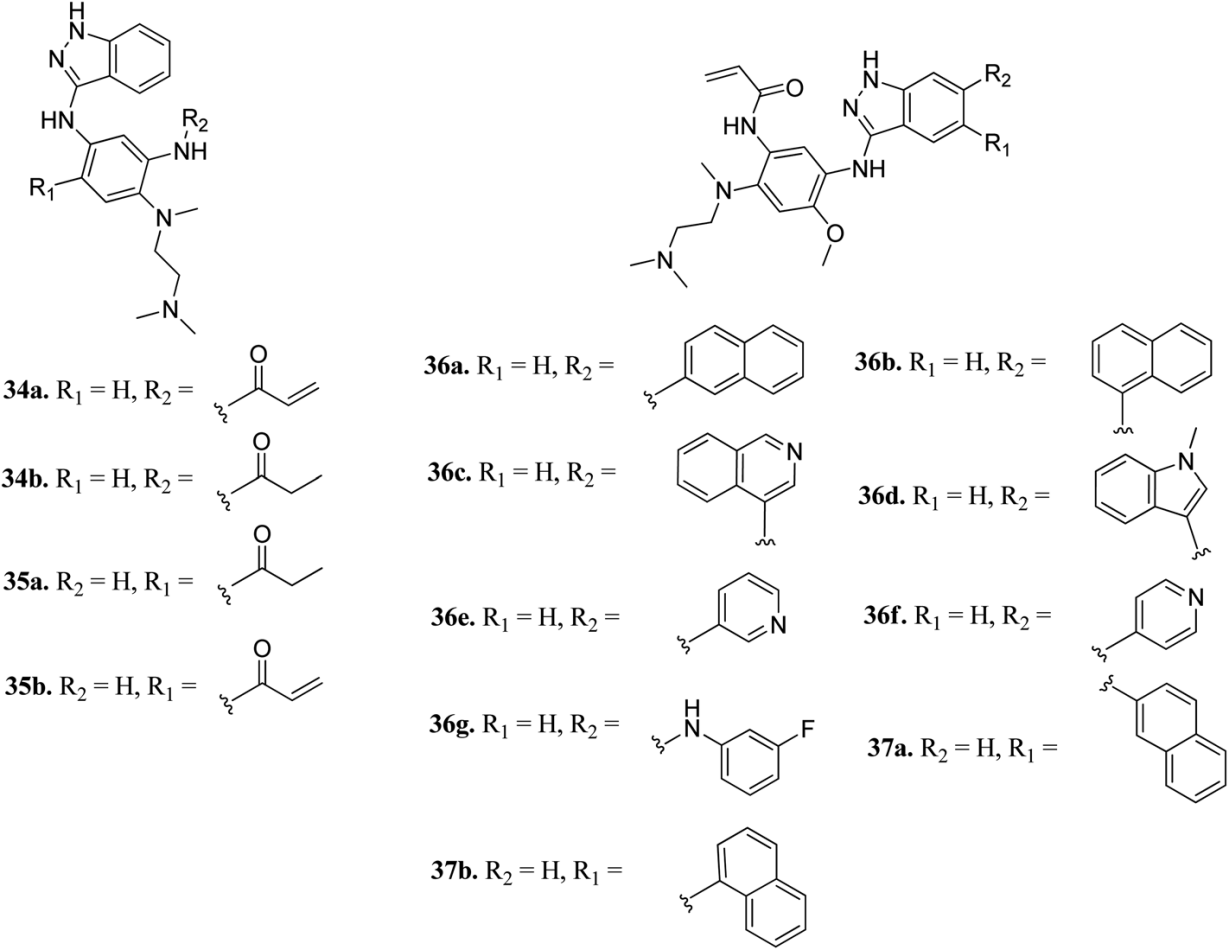
Indazole-based covalent inhibitors of epidermal growth factor receptor (EGFR).

**Fig. 13:**
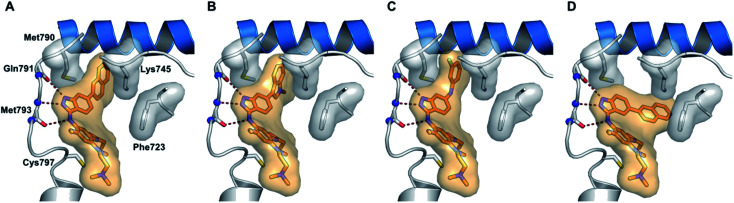
Proposed docking models of 36a (A), 36d (B), 36g (C) and 37a (D) with EGFR (reproduced with permission).

Tomassi *et al.* reported indazole-based covalent inhibitors (Fig. 12) of epidermal growth factor receptor (EGFR).^60^ Initial screening suggested that the derivatives with an acrylamide moiety (34a and 35a) had enhanced activity compared to their reversible counterpart (34b and 35b), respectively. Further, 35a was found to be selective for the EGFR wild type with IC_50_ = 3352 and 462 nM against the L858R and L858R/T790M mutants, respectively. The introduction of a 1-naphthyl substituent (36b and 37a, IC_50_ = 1–4.3 nM and 1.1–8.1 nM, respectively) resulted in good activity with single-digit nM IC_50_ values compared to the 2-naphthylsubstituted derivatives (36a and 37b, IC_50_ = 25–312 nM and 2.7–33 nM, respectively) with better activity against human epidermoid carcinoma (A431), hepatocellular carcinoma (HCC827) and lung adenocarcinoma (H1975) cancer cell lines. Substitution with different heterocycles such as isoquinolin-4-yl and *N*-methylindol-3-yl resulted in better activity against EGFR, L858R and L858R/T790M mutants (36c and d, single-digit nM IC_50_ values) than the pyridine derivatives (36e and f, double-digit nM IC_50_). Interestingly, the fluorinated derivative (36g) showed remarkable activity in the sub-nanomolar range against all variants of EGFR and EC_50_ values of 191 and 22 nM for H1975 and HCC827, respectively. Further, the docking models of 36a, 36d, 36g and 37a with EGFR supported the observed activity of these compounds (Fig. 13).

### (iv) Interleukin-2-inducible T-cell kinase (ITK) inhibitors

Interleukin-2-inducible T-cell kinases (ITKs) are S member of the Tec family tyrosine kinases, which play a crucial role in the intracellular signaling of hematopoietic cells.^61^ Studies have established that a deficiency in ITK results in the suppression of cell proliferation,^62–64^ and abnormal values of ITK result in T-cell malignancies.^65,66^

**Fig. 14:**
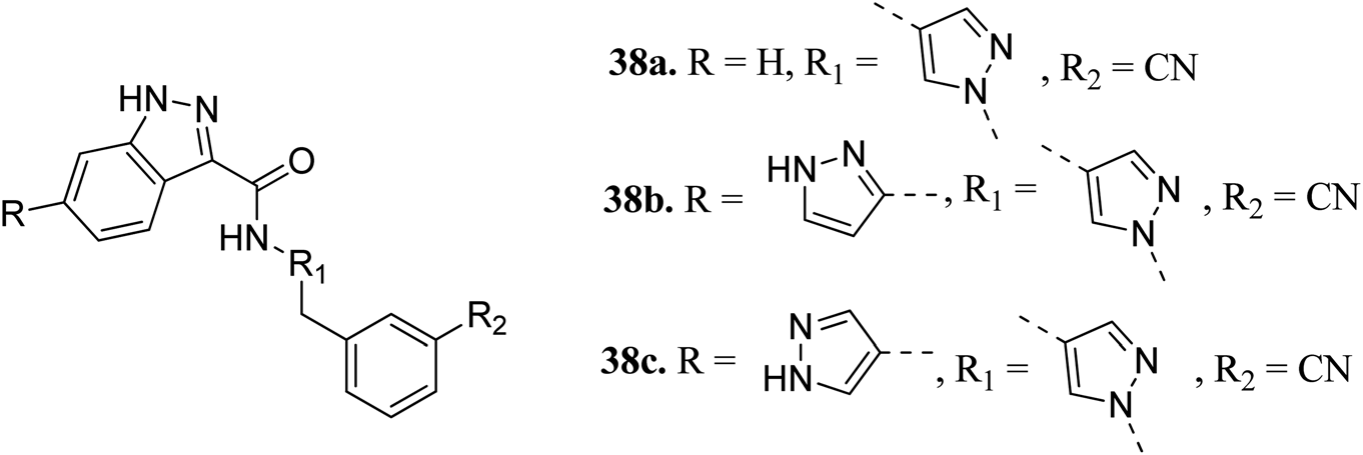
Optimization of indazole derivatives as ITK inhibitors.

Heifetz *et al.* optimized indazole derivatives (Fig. 14) as interleukin-2-inducible T-cell kinase (ITK) inhibitors.^67^ Compound 38a (PIE energy = −79.88 kcal mol; ITK *K*_i_ = 43 nM) was taken as the lead compound for further optimization to discover 38b and 38c. The pair interaction energy (PIE) calculated using the fragment molecular orbital (FMO) method for 3k (−107.80 kcal mol^−1^) was found to be better than 38b (−97.97 kcal mol^−1^). The same trend was reflected by the calculated ITK *K*_i_ values, which were 7.8 nM for 38b and 0.8 nM for 3bc compared to 43 nM for 38a.

### (v) pan-Tropomyosin receptor kinase (pan-Trk) inhibitors

Tropomyosin receptor kinases (TRKs) belong to the family of cell surface receptor tyrosine kinase (RTK) and are further classified into TRKA, TRKB and TRKC.^68^ Various studies reported that TRKs can mediate cell proliferation, metabolism, differentiation and apoptosis.^69–71^ The abnormal expression of TRK can lead to various types of human cancers.^72–75^

**Fig. 15:**
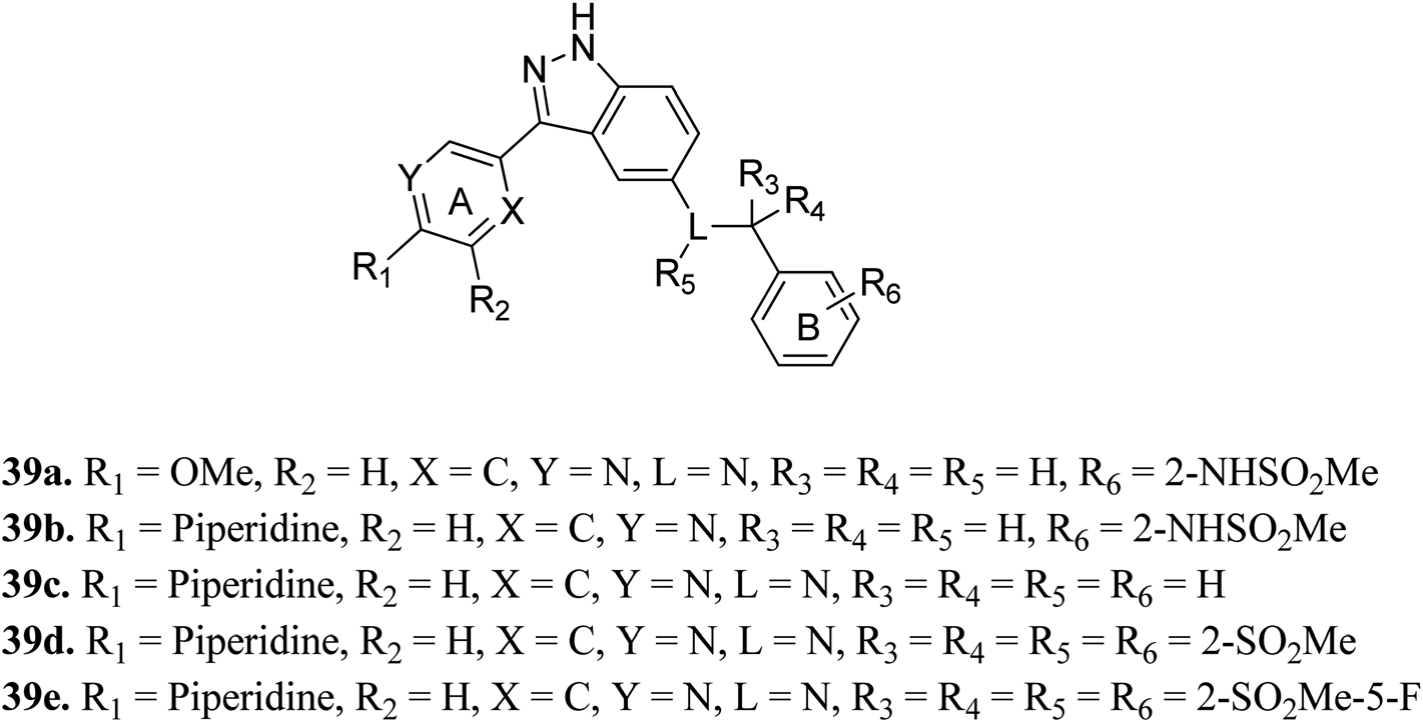
3-Aryl-indazole derivatives as inhibitors of pan-Trk.

Shirahashi *et al.* reported 3-aryl-indazole derivatives as inhibitors (Fig. 15) of pan-Trk for the treatment of pain.^76^ Among the synthesized compounds by varying the substitution in ring A, compound 39a with a methoxypyridine moiety was found to exhibit the highest potency but moderate metabolic stability. Replacement of the methoxy group with piperidine (39b) led to high cell permeability and significant potency (TRKA IC_50_ = 1.6 nM). Further optimization of 39b suggested that the non-branched nitrogen linker with phenyl ring B substituted at the 2-position by a sulfonamide group (39d) together with a fluoro group at the 5-position (39e) exhibited the highest cell permeability together with excellent potency among the synthesized compounds with TRKA IC_50_ values of 1.2 nM and 0.3 nm, respectively, compared to parent derivative 39c with TRKA IC_50_ = 73 nM. Docking studies against AZ-23 bound to the TRKA protein also supported the higher binding affinity of 39e compared to 39d followed by 39b.

### (vi) Glycogen synthase kinase 3 (GSK-3) inhibitors

Glycogen synthase kinase-3 (GSK-3) inhibitors are a class of serine/threonine kinases and classified further into the GSK-3α and GSK-3β forms.^77^ Further, GSK can act as a tumor suppressor in many cancer types such as lung,^78,79^ breast,^80–82^ skin,^83,84^ pancreas,^85–88^ hepatocellular carcinoma^89,90^ and leukemia.^91,92^ However, to date, no USFDA-approved GSK inhibitor is available in the market for the treatment of cancer.

**Fig. 16:**
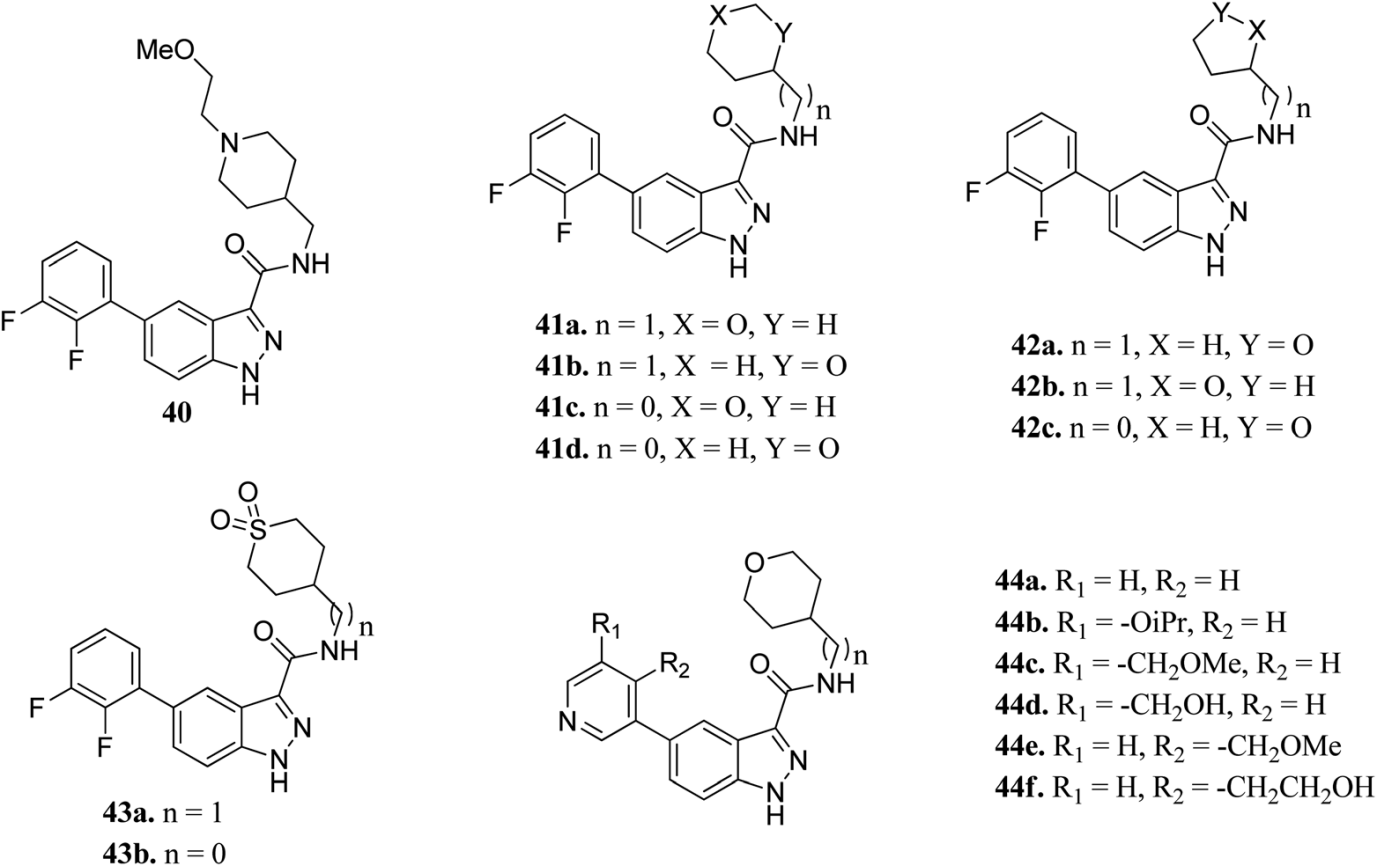
Indazole derivatives with improved GSK selectivity.

Prati *et al.* reported the structure–activity relationship of compounds (41–44) derived from 40*via* the introduction of different groups around the 1*H*-indazole-3-carboxamide scaffold for improving GSK selectivity (Fig. 16).^93^ Their studies suggested that the replacement of the (2-methoxyethyl)-4-methylpiperidine group by oxanyl, oxolanyl, thiolanedionyl and thianedionyl groups reduced the lipophilicity of the resultant derivatives (33–41) and led to improved selectivity towards the human ether-*à*-go-go-related dene (hERG) by 41–500 fold compared to compound 40. Further, replacement of the difluorophenyl moiety with polar pyridinyl and alkoxy-substituted pyridinyl groups led to compounds (44a–f) with promising activities (hERG IC_50_ > 40 μM), except for compounds 44b and 44c (hERG IC_50_ = 0.86 and 5.70 μM, respectively). Compound 44d emerged as the most promising compound with excellent activity (GSK-3β; IC_50_ = 0.004 μM and hERG; IC_50_ > 100 μM).

**Fig. 17:**
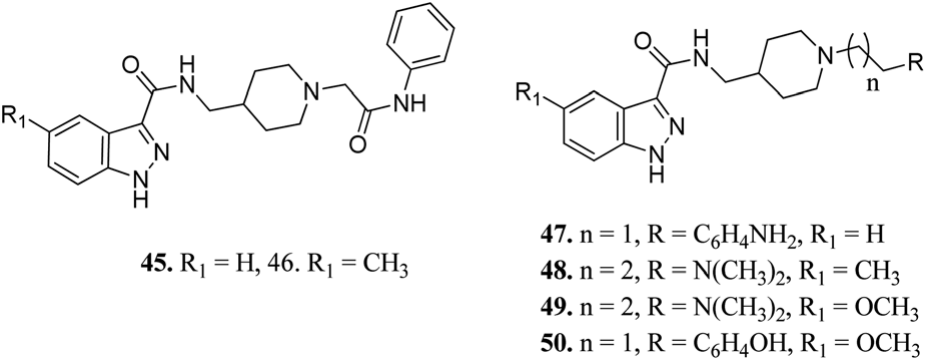
1*H*-Indazole-3-carboxamide derivatives as potential inhibitors of human GSK-3.

Ombrato *et al.* reported the structure-based discovery of 1*H*-indazole-3-carboxamide derivatives (Fig. 17) as potential inhibitors of human GSK-3.^94^ These compounds were found to bind to the ATP binding site of GSK-3β. Substitution of the amine group with dimethylamine did not lead to any improvement in activity (47 and 8). The activity of compound 48 with a methyl group at the 5-position of the indazole ring was found to be lower than that of methoxy derivatives 49 (IC_50_ = 1.7 μM) and 50 (IC_50_ = 0.35 μM), suggesting the importance of this group for high potency. The addition of a methyl group on the indazole phenyl ring of 45 (IC_50_ = 3.0 μM) led to a remarkable increase in activity (46, IC_50_ = 0.64 μM).

**Fig. 18:**
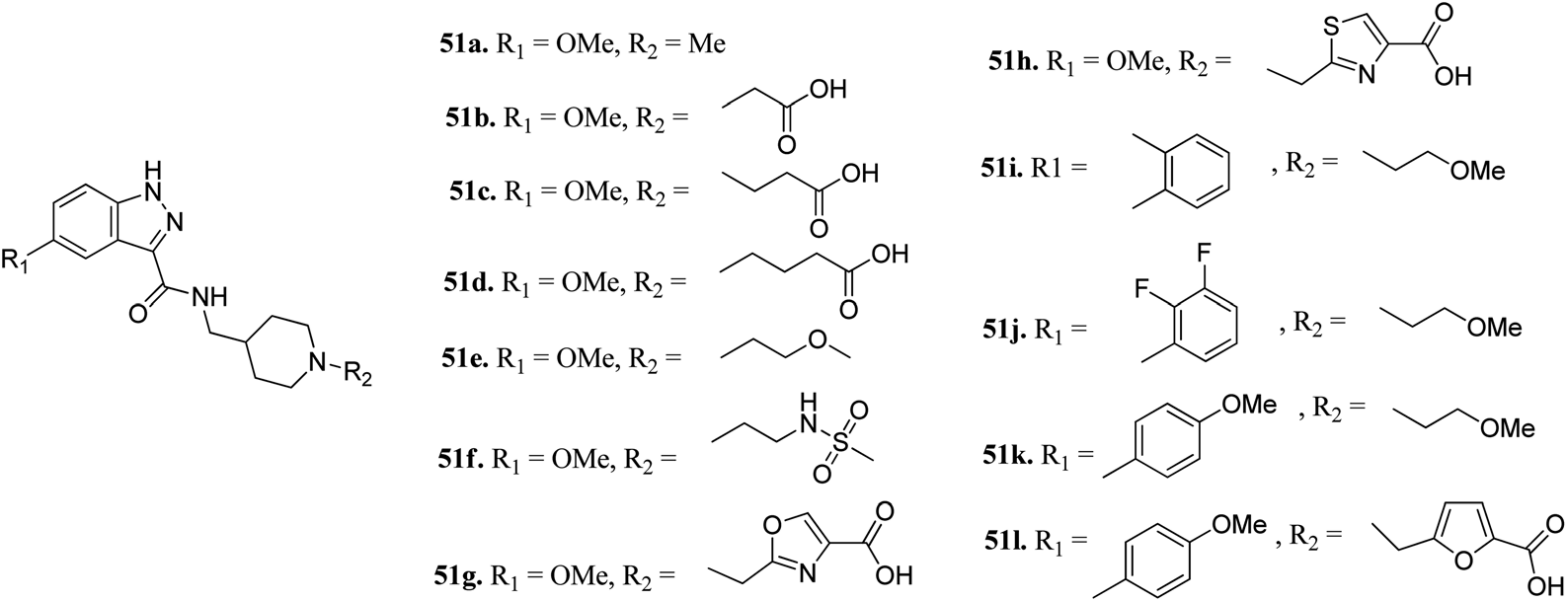
5-Substituted-*N*-(piperidin-4-ylmethyl)-1*H*-indazole-3-carboxamide as glycogen synthase kinase-3 (GSK-3) inhibitors.

Furlotti *et al.* reported 5-substituted-*N*-(piperidin-4-ylmethyl)-1*H*-indazole-3-carboxamide derivatives (Fig. 18) as glycogen synthase kinase-3 (GSK-3) inhibitors.^95^ Replacement of the methyl group on the piperidine ring of 51a (IC_50_ = 1.20 μM) with *N*-alkylcarboxylic acid led to an increase in activity (51b, 51c and 51d; IC_50_ = 0.67, 0.69 and 0.23 μM, respectively), whereas the introduction of a terminal polar group did not alter its activity (51e and 51f; IC_50_ = 0.87 and 1 μM, respectively). Interestingly, carboxylic acid derivatives 51g and 51h exhibited remarkable activity of 0.07 and 0.05 μM, respectively. Further optimization led to 2,3-difluorophenyl derivative 51j with IC_50_ = 18 nM, whereas the 2-methyl derivative (51i) and 4-methoxy phenyl derivatives (51k and 51l) exhibited poor activity. Further studies showed that 51j exhibited good *in vivo* PK activity and was found to be selective against only 34 kinases with more than 50% inhibition in a study for 216 kinases.

### (vii) Aurora kinase (AURK) inhibitors

Aurora kinases (AURKs) are a class of serine/threonine kinases, which can further be classified into AURKA, AURKB and AURKC.^96^ Overexpression of the aurora kinases has been observed in many cancer types such as breast cancer,^97–100^ gastrointestinal cancer,^101,102^ ovarian cancer^103–105^ and other tumors,^106–108^ thereby making these kinases important therapeutic targets to develop selective anticancer agents.

**Fig. 19:**
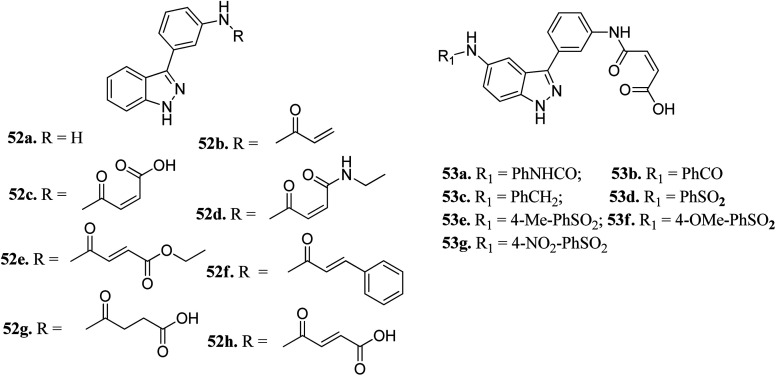
Novel indazole derivatives as inhibitors of Aurora kinases.

Chang *et al.* reported novel amide derivatives of indazole as inhibitors (Fig. 19) of Aurora kinases.^109^ Compound 52a was screened as the initial hit with an IC_50_ value of 13 μM. Introduction of an acryloyl moiety enhanced the kinase activity of the resulting compounds (52b and 52c with IC_50_ = 1.66 and 0.79 μM, respectively), whereas the ethylamide derivative (52d) was found to be inactive. Substitution of indazole at the C5 or C6 position with phenyl urea (53a), phenyl amide (53b) and benzylamine (53c) resulted in either the same or better activity with IC_50_ < 1 μM. Interestingly, the C5-substituted sulfonamide derivative (53d) exhibited remarkable activity (IC_50_ = 26 nM). Further substitution of the phenylsulfonyl group with methyl (53e), methoxy (53f) and nitro (53g) groups resulted in equipotent derivatives, whereas substitution with a bulkier group led to a decrease in activity.

**Fig. 20:**
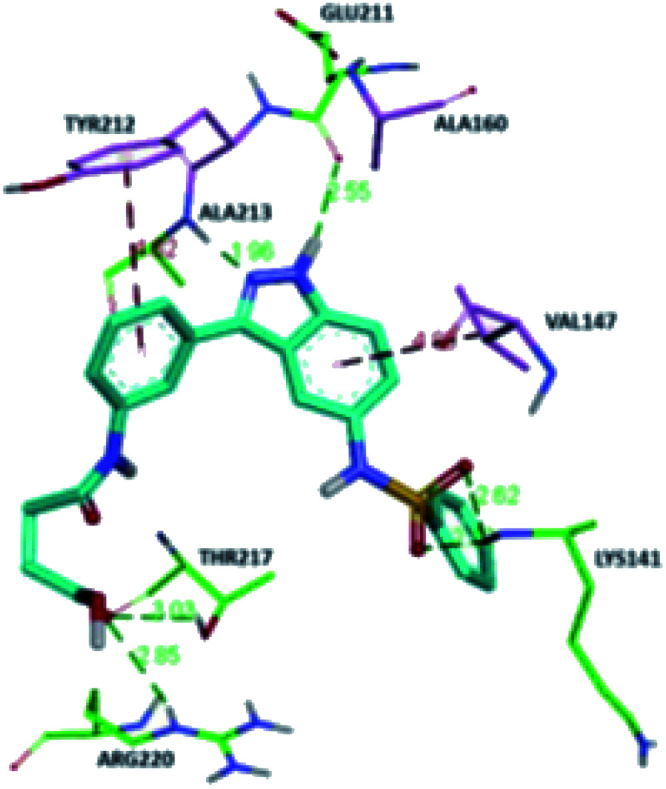
Docking studies of 53d with Aurora A (PDB ID: 2W1C). Green lines represent hydrogen bonding and red lines represent hydrophobic interactions (reproduced with permission; License Number: 5087990401536).

**Fig. 21:**
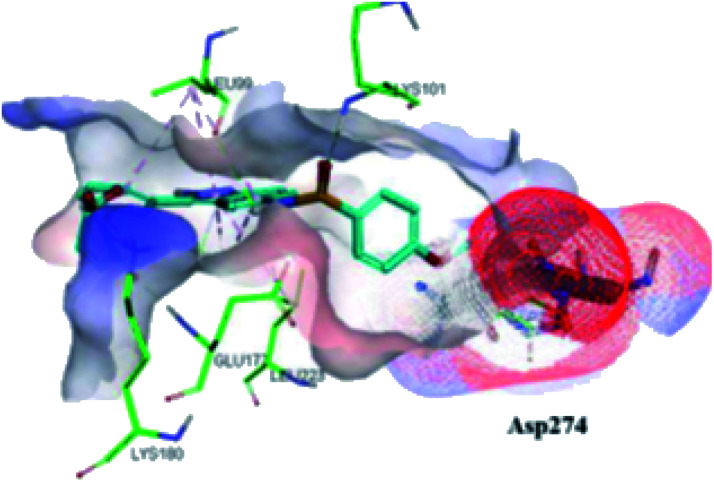
Docking studies of 53f with Aurora B (PDB ID: 4C2V). Green lines represent hydrogen bonding and red lines represent hydrophobic interactions (reproduced with permission; License Number: 5087990401536).

The molecular docking analysis of 53d suggested the binding of indazole with hinge residues of Glu211 and Ala213, whereas the sulfonyl group formed a hydrogen bond with Lys141, which contributed its significant potency against Aurora A. In addition, the carboxylic acid of 53d formed a hydrogen bond with Thr217 and Arg220 (Fig. 20). Conversely, the methoxy group of 53f (Fig. 21) offered steric hindrance to Asp274 in the back pocket of Aurora A, resulting in lower activity.

**Fig. 22:**
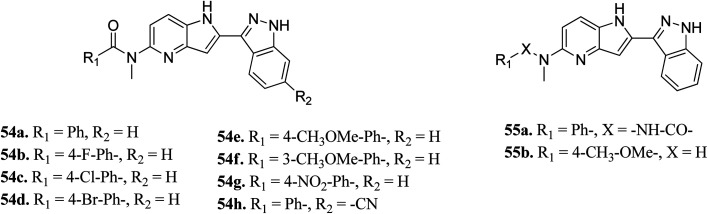
3-(Pyrrolopyridin-2-yl)indazole derivatives as inhibitors of Aurora kinase A.

Song *et al.* reported 3-(pyrrolopyridin-2-yl)indazole derivatives (Fig. 22) as inhibitors of aurora kinase A.^110^ The synthesized compounds were studied against a human hepatocarcinoma cancer cell line (SMMC-7721), human colorectal carcinoma cancer cell line (HCT116), human lung carcinoma cancer cell line (A549), and human leukemia cancer cell line (HL60). Besides urea derivatives 55a and 55b, which showed remarkable activities (IC_50_ = 0.0083–1.43 μM), all the other compounds were active against the tested cell lines with moderate potency. The introduction of electron-donating groups (54e; IC_50_ = 3.32–29.47 μM and 54f; IC_50_ = 1.42–22 μM) and electron-withdrawing group (54g, IC_50_ = 6.80–34.47 μM) led to a decrease in overall potency. Further studies on 54a, 54c and 55a against a set of 8 kinases suggested that these compounds act as inhibitors of Aurora kinase A with IC_50_ = 32, 46 and 519 nM, respectively, emphasizing the requirement of a halogen atom for enhanced potency. These studies were further supported by a docking study.

**Fig. 23:**
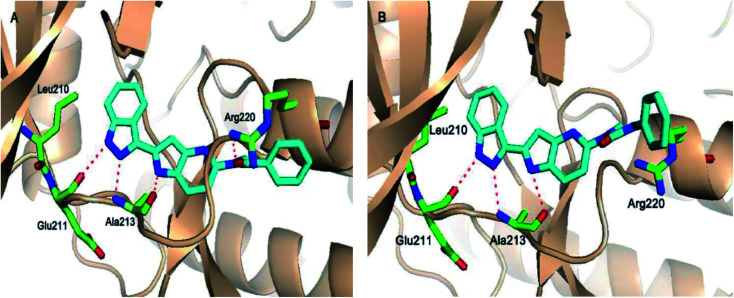
(A) Docking study of 54a with Aurora kinase A (reproduced with permission; License Number: 5087590256958). (B). Docking study of 55a with Aurora kinase A (reproduced with permission; License Number: 5087590256958).

Fig. 23 depicts the binding mode of 54a and 55a with Aurora kinase A. Both derivatives occupied the ATP binding site of Aurora kinase A, where the binding was significantly stabilized by hydrogen bonding with the hinge backbone of Ala213 and Glu211. The amide and urea linkage of 54a and 55a facilitated the orientation of the phenyl ring towards the solvent, respectively.

### (viii) pan-Pim inhibitors

pan-Pim kinases belong to the serine/threonine kinase family, which are further classified into three members including Pim-1, Pim-2 and Pim-3. Overexpression of these kinases is associated with various crucial processes such as cell proliferation, survival and differentiation.^111^ The expression level of Pim kinases varies based on the tumor type.^112^ An increase in Pim-1 and Pim-3 levels is a predictor of poor prognosis in gastric cancer.^113,114^

**Fig. 24:**
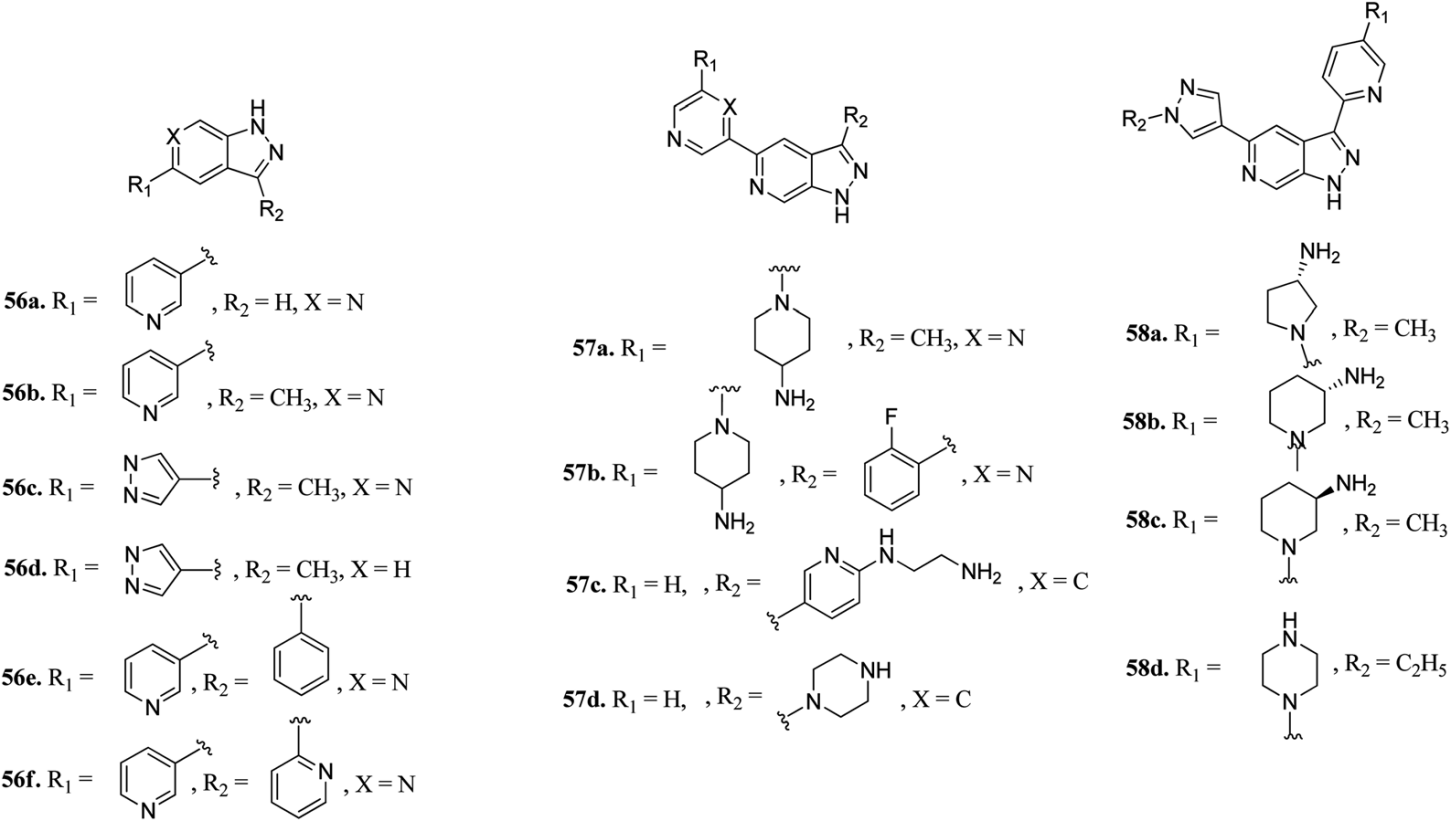
3,5-Disubstituted 6-azaindazoles as inhibitors of pan-Pim.

Hu *et al.* discovered 3,5-substituted-6-azaindazoles as inhibitors (Fig. 24) of pan-Pim kinase.^115^ The introduction of a methyl group at the C3-position of the azaindazole ring resulted in a 6-fold increase in the potency of compound 56a (*K*_i_ = 1.3–6 μM) to compound 56b (*K*_i_ = 0.2–0.8 μM) against Pim-1, Pim-2 and Pim-3. The presence of N6 in compound 56c resulted in 3–10-fold potency (*K*_i_ = 0.3–0.9 μM) as compared to compound 56d (*K*_i_ = 2.7–3.2 μM). Interestingly, replacement with a phenyl or pyridin-2-yl group resulted in a remarkable increase in potency (56e; *K*_i_ = 0.005–0.5 μM and 56f; *K*_i_ = 0.01–0.07 μM), respectively. Substitution at the C-3 position of the azaindazole ring with five- or six-membered heterocyclic rings (57a–d and 58a–c) led to moderate potency, respectively. Compound 58d exhibited the most balanced potency (*K*_i_ = 0.041–0.73 μM) and stability *in vivo*.

**Fig. 25:**
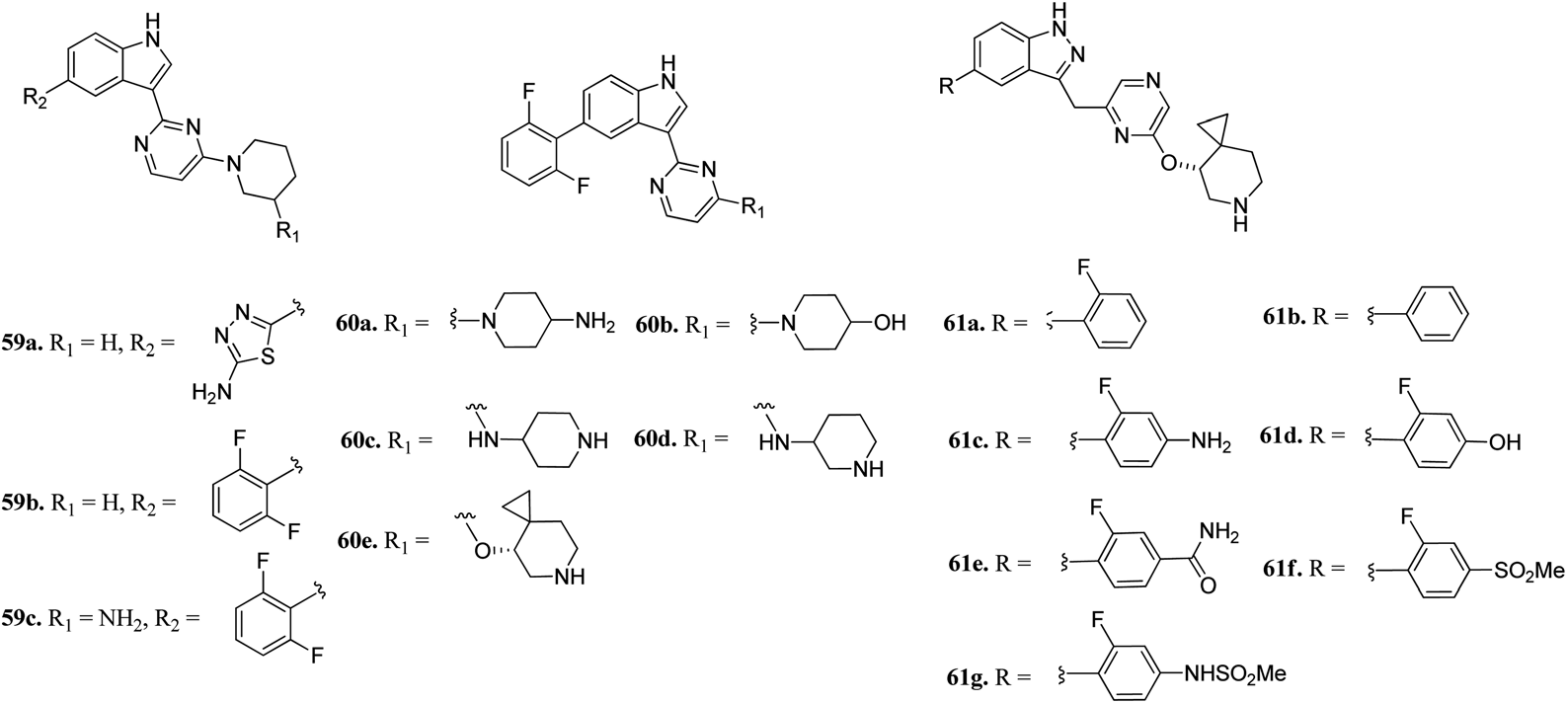
3-(Pyrazin-2-yl)-1*H*-indazole derivatives as potential inhibitors of pan-Pim kinase.

Wang *et al.* reported 3-(pyrazin-2-yl)-1*H*-indazole derivatives (Fig. 25) as potential inhibitors of pan-Pim kinase.^116^ Compound 59a showed good potency (Pim1-3, IC_50_ = 3–11 nM) but lacked metabolic stability, whereas compound 59b exhibited good metabolic stability but moderate potency (Pim1-3, IC_50_ = 142 ≥ 3000 nM). Replacement of the piperidine moiety of 2 with 2-aminopiperidine led to compound 59c with nanomolar potency (Pim1-3, IC_50_ = 3–70 nM). Replacement of 3-aminopiperidine with 4-piperidine led to a further improvement in activity (60a; IC_50_ = 1–9 nM), whereas replacement of the amine group with hydroxyl led to a decrease in activity (60b; IC_50_ = 44–362 nM). Interestingly, the spirocyclopropyl analogue (S-60e) exhibited remarkable activity (IC_50_ = 0.4–1.1 nM); however, moderate cellular potency against a human myeloma cancer cell line (KMS-12 BM) (IC_50_ = 1400 nM). Further, the removal of fluorine atoms from 60e led to a decrease cellular potency (IC_50_ = 1970 and 3400 nM for 61a and 61b, respectively). Interestingly, the introduction of polar groups at the C4 position of 2-fluorophenyl ring led to improved cellular potency (61c–g; KMS-12 BM, IC_50_ = 120–30 nM), while retaining enzyme activity.

### (ix) Plo-like kinase 4 (PLK4) inhibitors

Plo-like kinase 4 (PLK4) is a family of tyrosine serine/threonine kinases that play an important role in cell regulation and cellular response.^117,118^ An abnormal level of PLK4 results in tumorigenesis and centriole amplification and is further associated with several human cancers.^119–121^

**Fig. 26:**
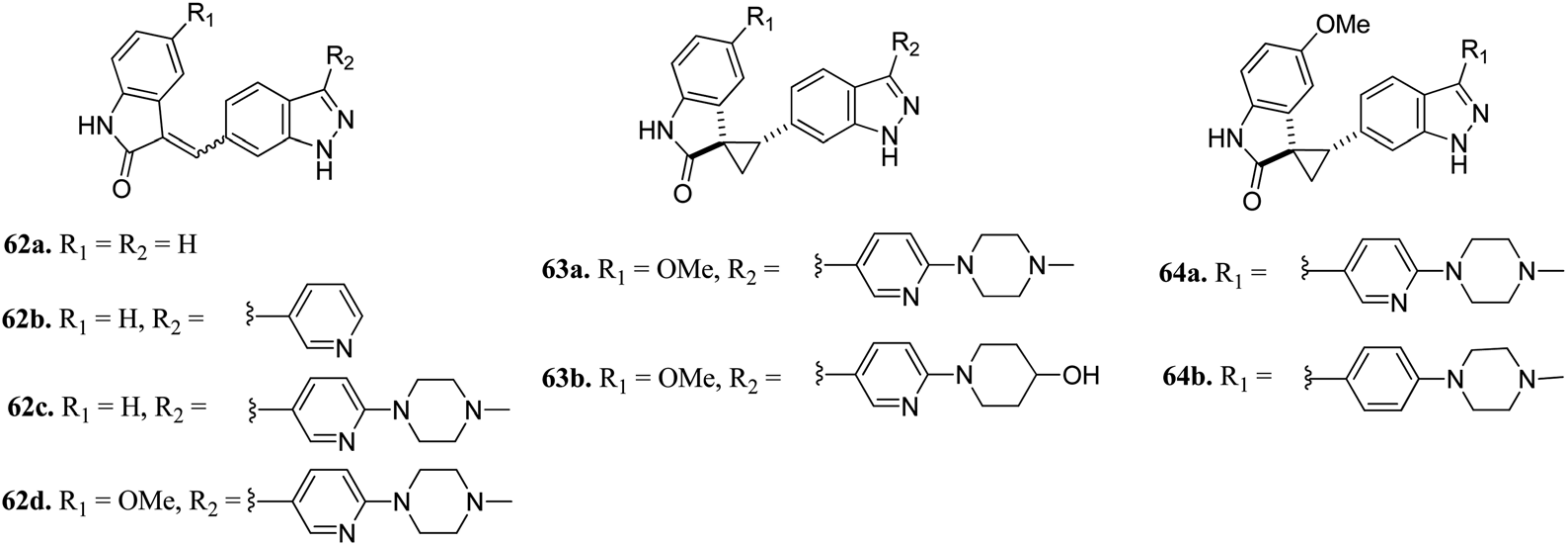
(3-Aryl-1*H*-indazol-6-yl)spiro[cyclopropane-1,3′-indolin]-2′-one derivatives as PLK4 inhibitors.

Li *et al.* reported (3-aryl-1*H*-indazol-6-yl)spiro[cyclopropane-1,3′-indolin]-2′-one derivatives (Fig. 26) as PLK4 inhibitors.^122^ The pyridin-3-yl analogue of the lead compound 62a possessed 10-fold potency (62b, IC_50_ = 0.029 μM and 0.027 μM against PLK4 and fms-like tyrosine kinase 3 (FLT3) *versus*62a, IC_50_ = 0.29 and 0.049 μM, respectively). Further, (4-methylpiperazin-1-yl)pyridin-3-yl (62c, IC_50_ = 0.029 μM and 0.027 μM against PLK4 and FLT3, respectively) and the methoxy derivative of 4f (62d, IC_50_ = 0.0024 μM and 0.028 μM against PLK4 and FLT3, respectively) showed significantly enhanced activity. Optimization of 62c led to compound 63a, which exhibited activity against FGFR in the nanomolar range (IC_50_ = 0.0010 nM) with 2-fold selectivity over FLT3 (IC_50_ = 0.26). Further optimization led to 64a and 64b having aryl substitution, which showed better activity compared to their vinyl counterpart 62c. Interestingly, among the 274 kinases studied, 64b was found to be selective to only 15 kinases with more than 50% inhibition.

### (x) Rho-associated coiled-coil kinase (ROCK) inhibitors

Rho-associated coiled-coil kinases (ROCKs) belong to the AGC family of serine/threonine protein kinases, which include ROCK1 and ROCK2. These play important roles in many cellular reactions such as cell proliferation, contraction, migration, adhesion and apoptosis,^123–125^ and therefore can be used as potential targets for cancer management.^126^

**Fig. 27:**
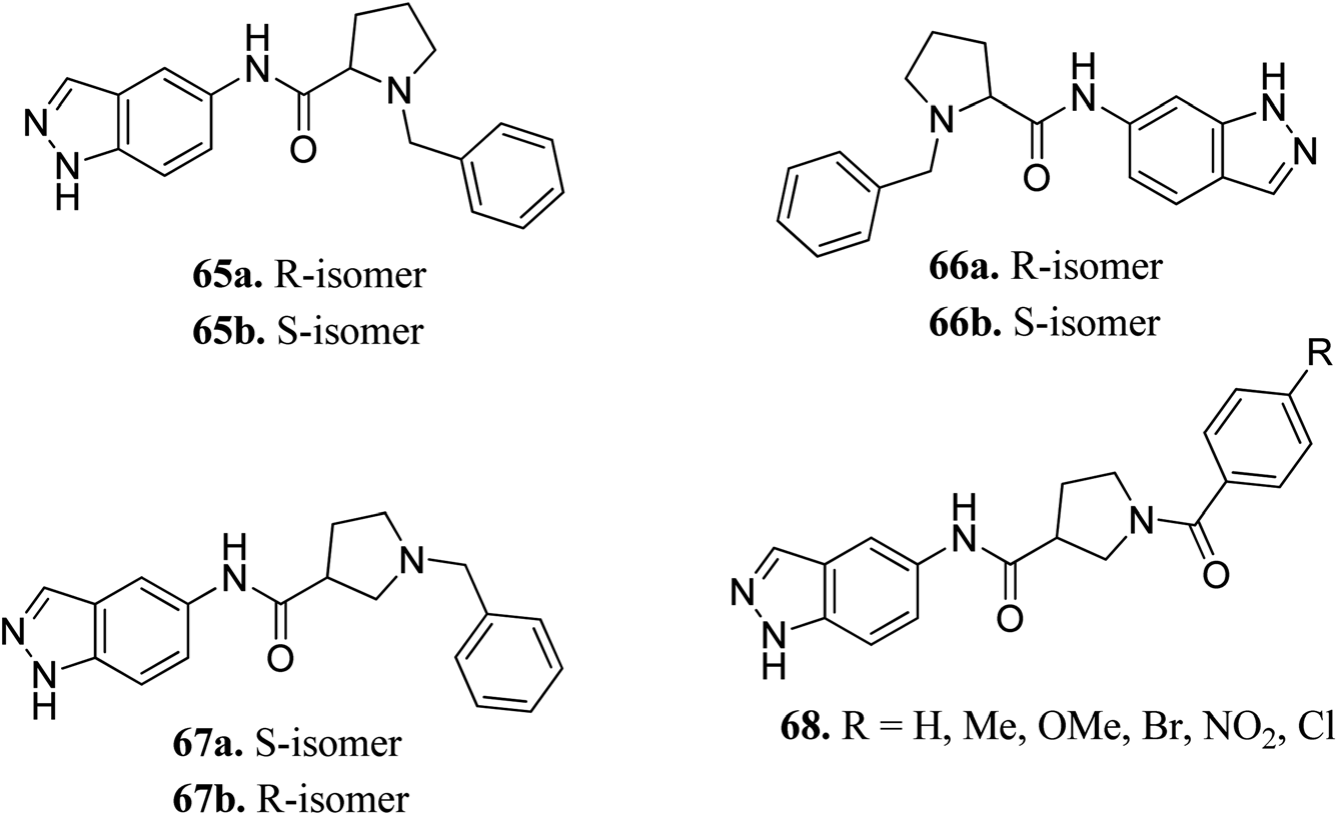
N-substituted prolinamido indazole derivatives as potent Rho-kinase inhibitors.

Yao *et al.* reported N-substituted prolinamido indazole derivatives (Fig. 27) as potent Rho-kinase inhibitors.^127^ The SAR studies indicated that derivatives with a β-proline moiety (67 and 68) having linear-shaped compounds possessed enhanced activity against ROCK I compared to the angular-shaped derivatives bearing an α-proline moiety (65 and 66). In the benzyl-substituted series (67), the substituent effect on the activity followed the order of CH_3_ > H > Br > OCH_3_ > F > NO_2_, CN. Further, the S-isomer (67a, IC_50_ = 0.42 μM) showed better activity than the R-isomer (67b, IC_50_ = 7.32 μM). Conversely, the R- and S-isomers exhibited similar activity in the case of 65 (65a, % inhibition = 24.3% *vs.*65b, % inhibition = 24.6%) and 66 (66a, % inhibition = 15.0% *vs.*66b, % inhibition = 23.7%).

**Fig. 28:**
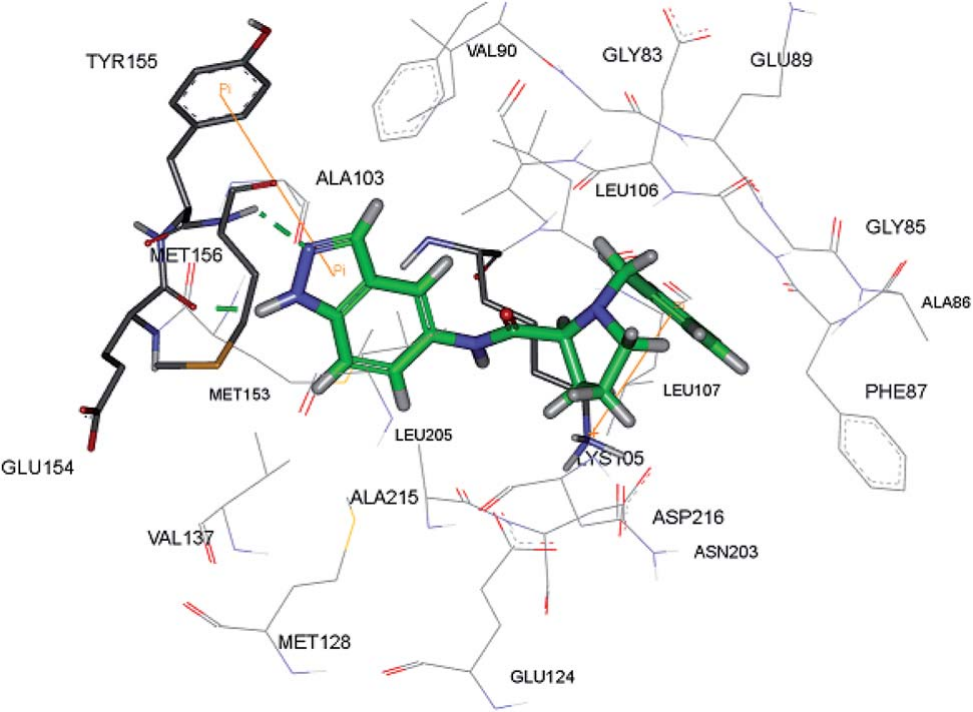
Proposed docking of 67 with the ATP binding pocket of ROCK1 (reproduced with permission).

The racemic mixture of 67 was also studied for its docking with ROCK1, which suggested hydrogen bond interactions between the N and NH of indazole with Met156. In addition, the terminal phenyl ring was also observed to participate in π-cation interactions with Lys105 (Fig. 28).

### (xi) CRAF inhibitors

CRAF belongs to the family of RAF kinases family of serine/threonine-specific kinases. It has been reported to play a role in oncogenic human cancers.^128,129^

**Fig. 29:**
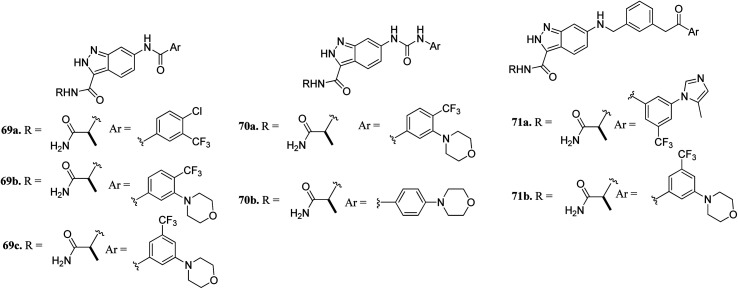
3-Carboxamido-2*H*-indazole-6-arylamide derivatives as selective CRAF inhibitors.

Aman *et al.* reported 3-carboxamido-2*H*-indazole-6-arylamide derivatives (Fig. 29) as selective CRAF inhibitors.^130^ 1,3,4-Substituted benzoic acid analogues with an amide linker exhibited good activity against human melanoma cell lines (WM3629 and A375P) (GI_50_ = 9.13 and 7.37 μM and 10.5 and 28.3 μM for 69a and 69b, respectively, *versus* GI_50_ = 30 μM for 69c). A similar trend was observed for 1,3,4-substituted benzoic acid derivatives with a urea linker (70a, GI_50_ = 3.51 and 10.05 μM *versus*70b, GI_50_ = 9.29 and 12.3 μM, respectively). Further optimization led to the discovery of bulkier and longer analogs 71a and 71b with remarkable activity (GI_50_ = 0.65 and 2.25 μM and 15.93 and 25.6 μM for 71a and 71b, against WM3629 and A375P, respectively). Moreover, 71a exhibited 35-fold higher activity against the WM3629 cell line and was found to be a selective CRAF inhibitor with an excellent selectivity profile and 99% inhibitory activity against CRAF as compared to 20% inhibitory activity against other kinases in the study with 32 kinases.

**Fig. 30:**
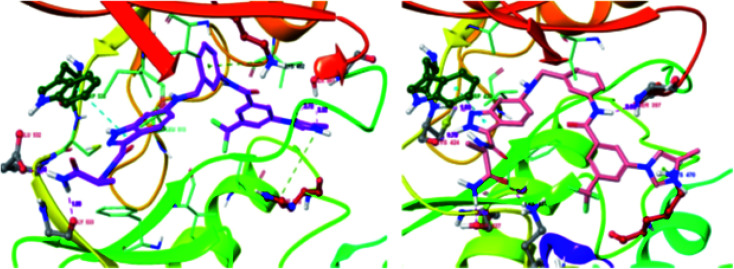
Docking structures of 71a with CRAF (3OMV) and BRAF V599E (reproduced with permission; License Number: 5087580376405).

These observations were further supported by the docking studies of 71a on CRAF (3OMV) and BRAF V599E (PDB Code: 1UWJ) (Fig. 30). These studies suggested that 71a was tightly bound to CRAF kinase with the formation of two hydrogen bonds of the nitrogen atoms of indazole and hinge residue Cys424. Two additional hydrogen bonds were also observed with the hinge residue of Trp423 and Lys431. In addition, a hydrogen bond between the amide of 71a bond and Ser357, π–π interaction between indazole and Trp423 and π-cation interactions between indazole and Lys470 were also observed. Conversely, only two hydrogen bond interactions of indazole with Gly533 and Ser 601 were possible in the case of the docking studies of 71a with BRAF V599E.

### (xii) Mitogen-activated protein kinase (MAPK) inhibitors

Mitogen-activated protein kinases (MAPKs) belong to the family of serine/threonine-specific protein kinases, which play a crucial role in cell growth, proliferation and survival.^131,132^

**Fig. 31:**
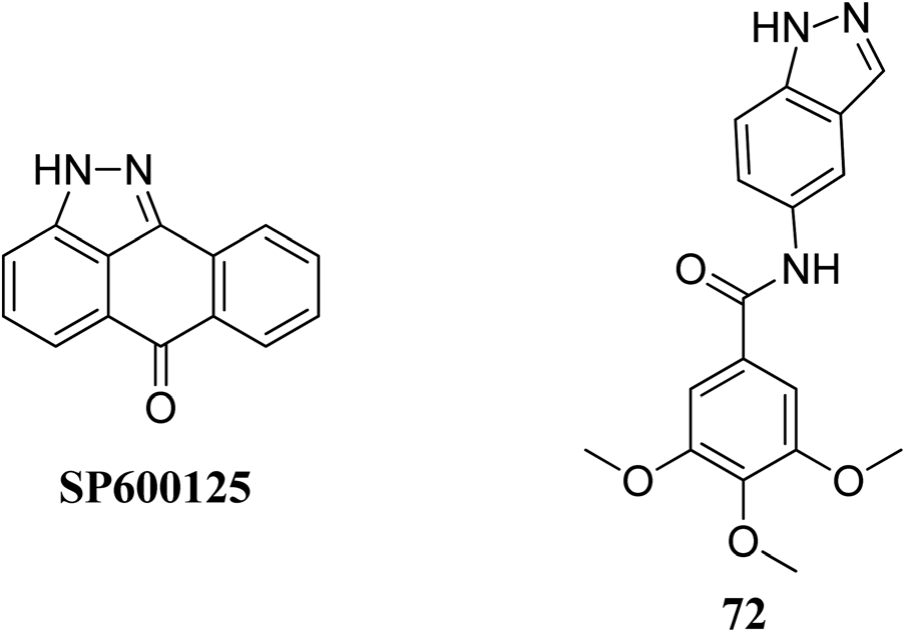
Galloyl benzamide-based indazole modulator of MAPK signaling pathways.

Leo *et al.* studied the effect of galloyl benzamide-based indazole derivatives (Fig. 31) on the MAPK signaling pathways in comparison to adenosine triphosphate (ATP)-competitive JNK inhibitor SP600125.^133^ Compound 72 was observed to be a poor inhibitor of P-gp and MRP1. Pretreatment with 72 at 1, 20 and 50 μM resulted in the inhibition of TNF-α-mediated phosphorylation of JNK, which was comparable to SP600125 at 20 μM and inhibited TNF-α-mediated p38 MAPK phosphorylation at high concentrations. A similar trend was also observed for the inhibition of the TNF-α-mediated phosphorylation of c-Jun N-terminal kinase, whereas no significant inhibition was observed in the case of ERK1/2, NF-κB for compound 72.

### (xiii) Janus kinase (JAK) inhibitors

The Human JAK family can be classified into 4 members including JAK1, JAK2, JAK3 and Tyk2.^134,135^ JAK2 is known to mediate important physiological functions such as cell proliferation.^136,137^ Overexpression of JAK3 is also associated with juvenile myelomonocytic leukemia.^138^ Tofacitinib was the first approved JAK inhibitor, after which various JAK inhibitors were developed.

**Fig. 32:**
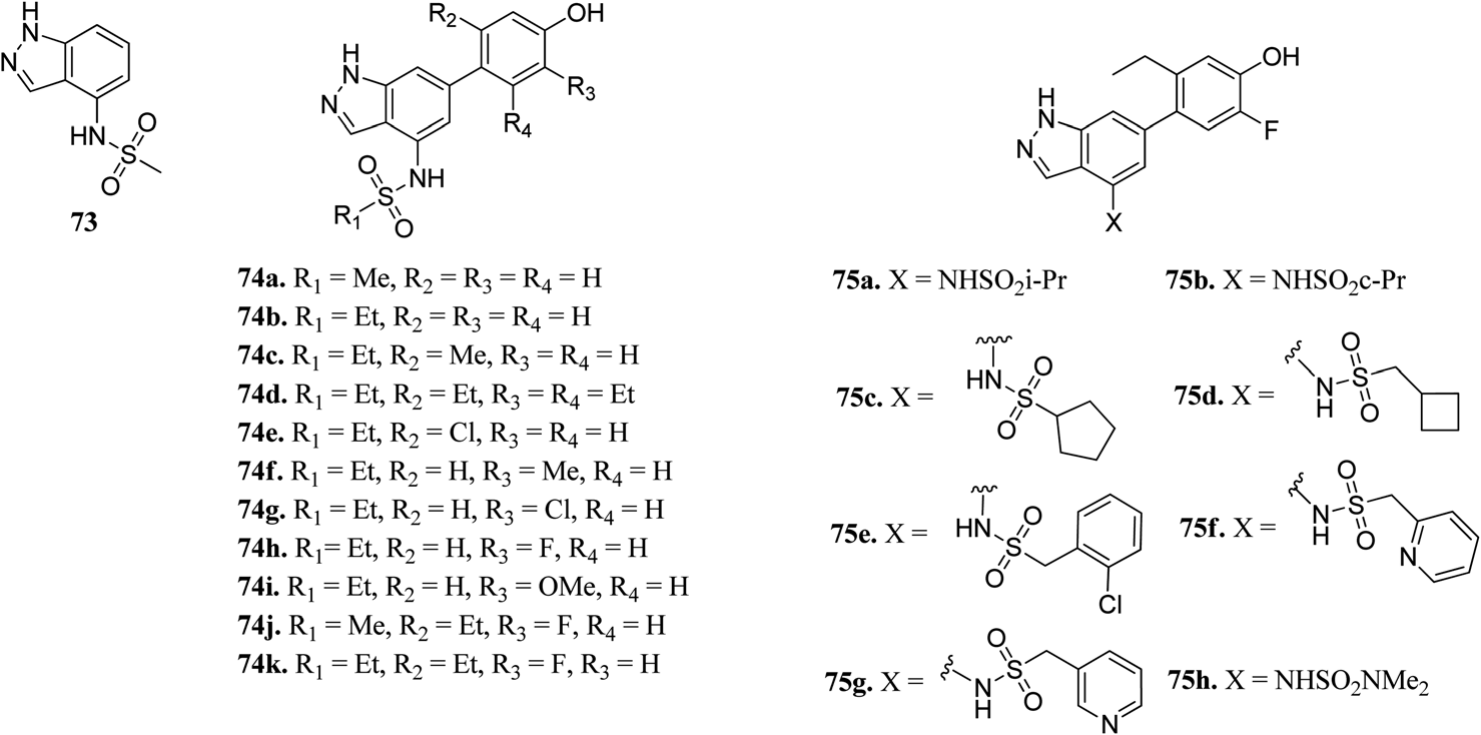
6-Arylindazole derivatives as JAK inhibitors.

Ritzen *et al.* reported 6-arylindazole derivatives (Fig. 32) as JAK inhibitors.^139^ The introduction of a phenolic group in indazolo fragment 73 led to an increase in potency in 74a (pIC_50_ = 5.4 *versus* 4.5). Replacement of the methylsulfonamide group with ethylsulfonamide led to a further increase in potency (74b, pIC_50_ = 5.7). Substitution of R_2_ with a small lipophilic group (74c and e) resulted in enhanced cellular potency, with 74d being the most active. Substitution at R_3_ with a halogen or alkoxy group (74f–74i) led to a slight increase in cellular potency with the halide analogs (74g–h) showing highest the potency in the group. Further optimization led to the discovery of 74i–j, with 74k as the most active compound in the series (pIC_50_ = 6.77) with good LLE values.

**Fig. 33:**
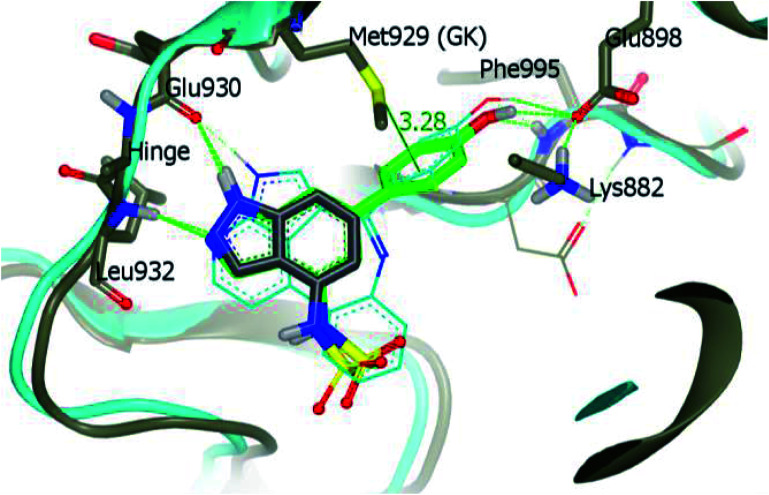
Proposed docking of 74a with JAK2 (reproduced with permission).

Derivative 74a was subjected to docking studies with JAK2, which suggested hydrogen bonding with Leu932 and Glu930. Further, additional hydrogen bonding was observed for the phenol group with αC helix residue Glu898 and NH group of Phe995. Also, the phenol group was found to be in contact with Met929 (Fig. 33).

**Fig. 34:**
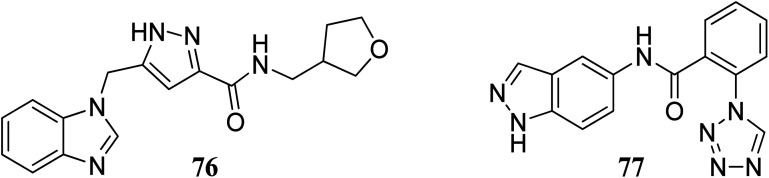
Structure-based virtual screening of indazole derivatives as potential inhibitors of Janus kinase (JAK).

Bajusz *et al.* reported the structure-based virtual screening of indazole derivatives (Fig. 34) as potential inhibitors of Janus kinase (JAK).^140^ The screened compounds 76 and 77 were found to be selective to the JAK2 subtype compared to the JAK1 subtype. Compound 77 exhibited remarkable activity against JAK2 (IC_50_ = 2.38 μM) compared to JAK1 (IC_50_ = 33 μM), whereas compound 76 exhibited IC_50_ = 5.72 μM with only 1% inhibition of JAK1 at 20 μM. Further, docking studies supported that the tetrazole ring of 77 was buried deep in the binding pocket of JAK2, whereas it is exposed towards the solvent in the case of JAK1.

### (xiv) Melanin-concentrating hormone receptor 1 (MCHR1) inhibitors

Melanin-concentrating hormone receptor 1 is reported to activate extracellular signal-regulated kinase pathways.^141^

**Fig. 35:**
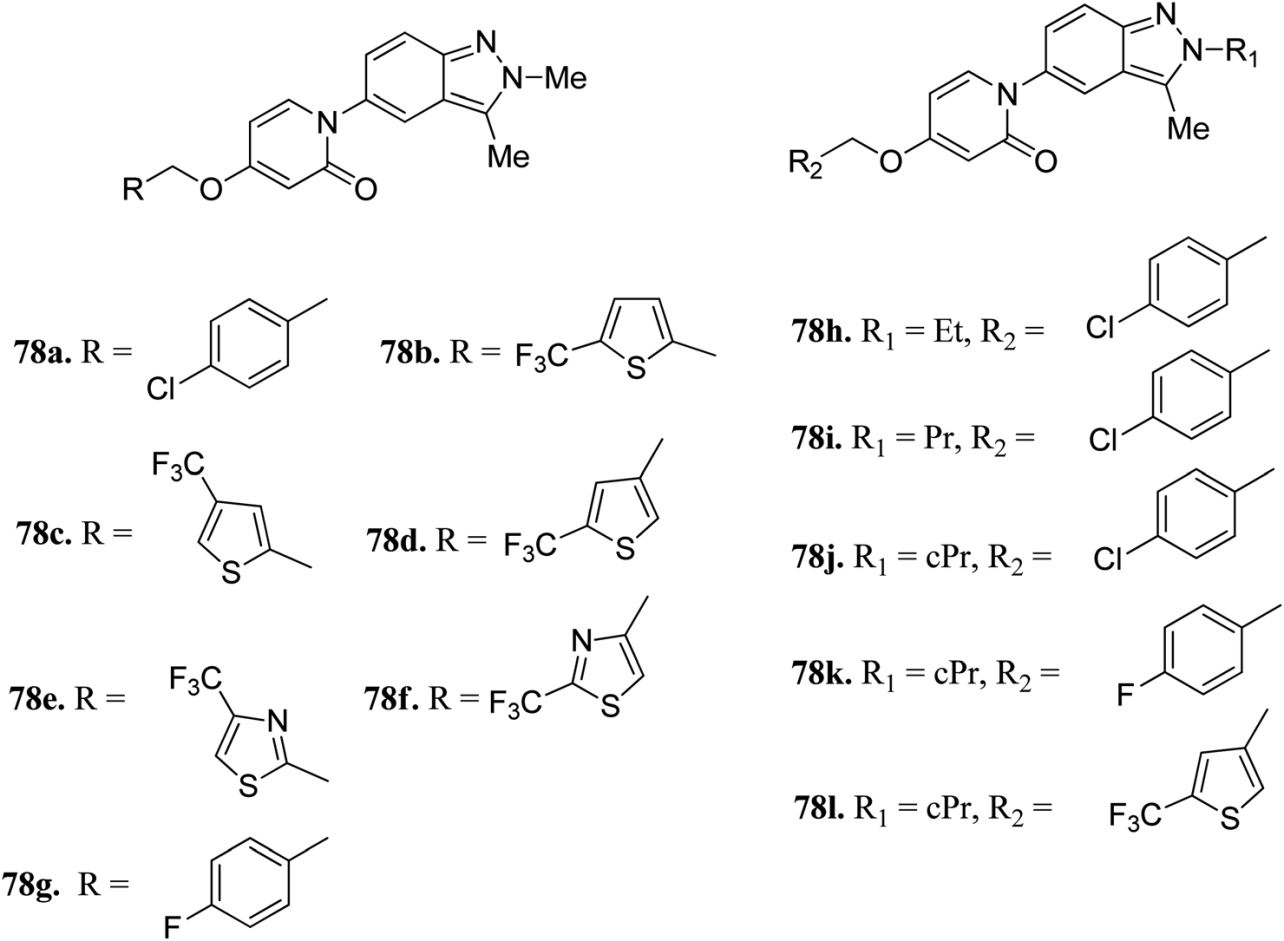
1-(2*H*-Indazole-5-yl)pyridin-2(1*H*)-one derivatives as MCHR1 antagonists.

Igawa *et al.* reported 1-(2*H*-indazole-5-yl)pyridin-2(1*H*)-one derivatives (Fig. 35) as melanin-concentrating hormone receptor 1 (MCHR1) antagonists.^142^ The structure–activity relationship (SAR) studies revealed that the 2,4-disubstituted thiophene derivatives (78c and d) were more potent than the 2,5-disubstituted thiophene (78b) and thiazole derivatives (78e and f). Interestingly, the 2-cyclopropylindazole derivatives (78j–l) possessed better activity than the methyl analogs (78a, 78g and 78d). Also, 78j (IC_50_ = 33 nM) and 78l (IC_50_ = 79 nM) were found to have the best *in vitro* potency and did not include the risk of mutagenesis. Derivative 78l also exhibited a superior anorectic effect in the *in vivo* studies and possessed good brain exposure.

### (xv) G protein-coupled receptor kinase 2 (GRK2) inhibitors

G protein-coupled receptor kinases (GRKs) are known to act as negative regulators of G protein-coupled receptors (GPCR) activity, and therefore play a crucial role in cell progression by controlling various physiological reactions such as cell proliferation, invasion, migration, apoptosis and tumor vascularization.^143–145^

**Fig. 36:**
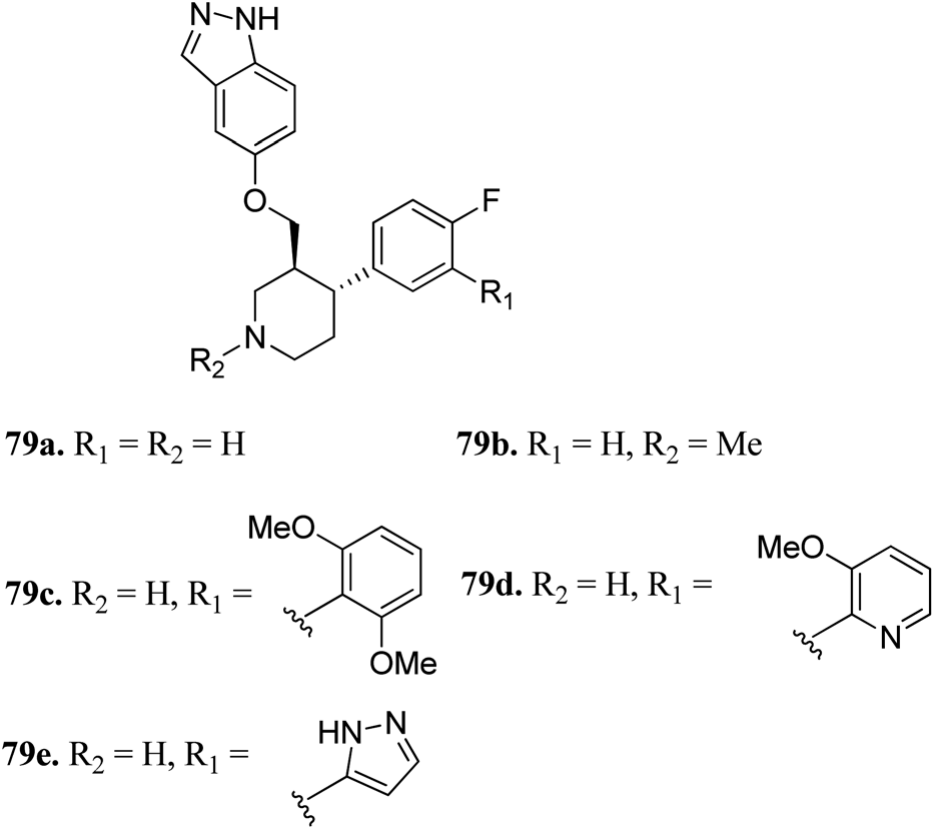
Indazole-paroxetine hybrids as G protein-coupled receptor kinase 2 inhibitors.

Bouley *et al.* reported the activity of indazole-paroxetine hybrids (Fig. 36) against G protein-coupled receptor kinase 2 (GRK2).^146^ Compound 79a exhibited 20-times greater potency (IC_50_ = 66 nM) than paroxetine (79b; IC_50_ = 1.38 μM) against GRK2 with an increase in the activity of 79a against GRK1, 5PKA, and ROCK1 and decrease in selectivity (5-fold) against ROCK1 compared to paroxetine (50-fold). The introduction of the 2,6-dimethoxybenzylamide group on fluorophenyl C-ring (79c) resulted in increased selectivity against GRK2 compared to other GRK and PKAs. Interestingly, the introduction of the 3-pyrazolylmethylamide group induced 79e, which was most the effective against GRK2 with an IC_50_ value of 8 nM and selective (20-fold) for other GRKs and PKAs. Conversely, *N*-methylation of the pyridine ring (79b) led to a decrease in potency against GRK1, GRK5 and PKA.

### (xvi) Extracellular signal-regulated kinase (ERK) inhibitors

Activation of the extracellular signal-regulated kinase (ERK) pathway is well known to contribute to various types of cancers.^147,148^ Therefore, inhibition of the ERK pathway can play a crucial role in cancer treatment.

**Fig. 37:**
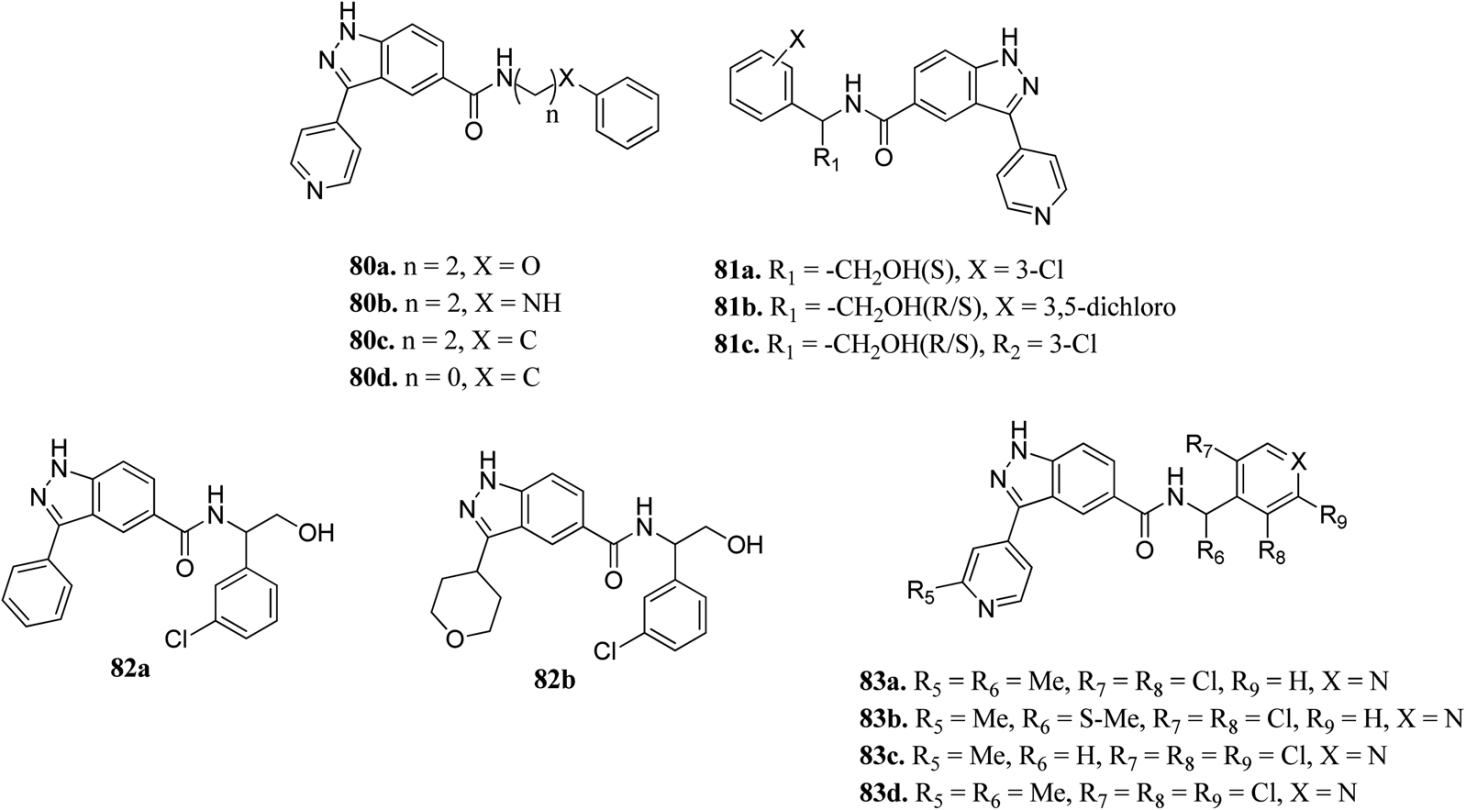
Indazole amide derivatives as inhibitors of extracellular signal-regulated kinase 1/2 (ERK1/2).

Li *et al.* reported indazole amide derivatives (Fig. 37) as inhibitors of extracellular signal-regulated kinase 1/2 (ERK1/2).^149^ Substitution of the lead compound 80a (IC_50_ = 63.3 nM) with nitrogen led to a decrease in activity (80b, IC_50_ = 139.5 nM), while replacement with carbon showed a positive effect (80c; ERK2, IC_50_ = 33.8 nM). Substitution of the phenyl ring with chlorine, and further shortening of the side chain resulted in 80d with IC_50_ = 12.6 nM. Interestingly, the S-isomer hydroxymethyl analogue (S-81a) exhibited remarkable activity (IC_50_ = 7.0 nM). Further, the introduction of pyridine at the benzylic position led to good enzyme activity with remarkable cellular activity (ERK2, IC_50_ = 16.7, 7.9, 13.2 nM and HT-29, IC_50_ = 0.35, 1.1, 0.713 nM for 83a, 83c and 83d, respectively). Further S-83b (ERK2, IC_50_ = 9.5 nM and HT-29, IC_50_ = 10.7) showed better enzyme activity but lower cellular activity than the R-isomer and racemate counterpart (83a).

### (xvii) Unc-51-like kinase 1 (ULK-1) inhibitors

Unc-51-like kinase 1 (ULK-1) is a cytoplasmic kinase that is involved in the autophagy process. It can either promote or inhibit tumor growth,^150^ and therefore either positive or negative regulation of ULK-1 can lead to cancer management.^151^

**Fig. 38:**
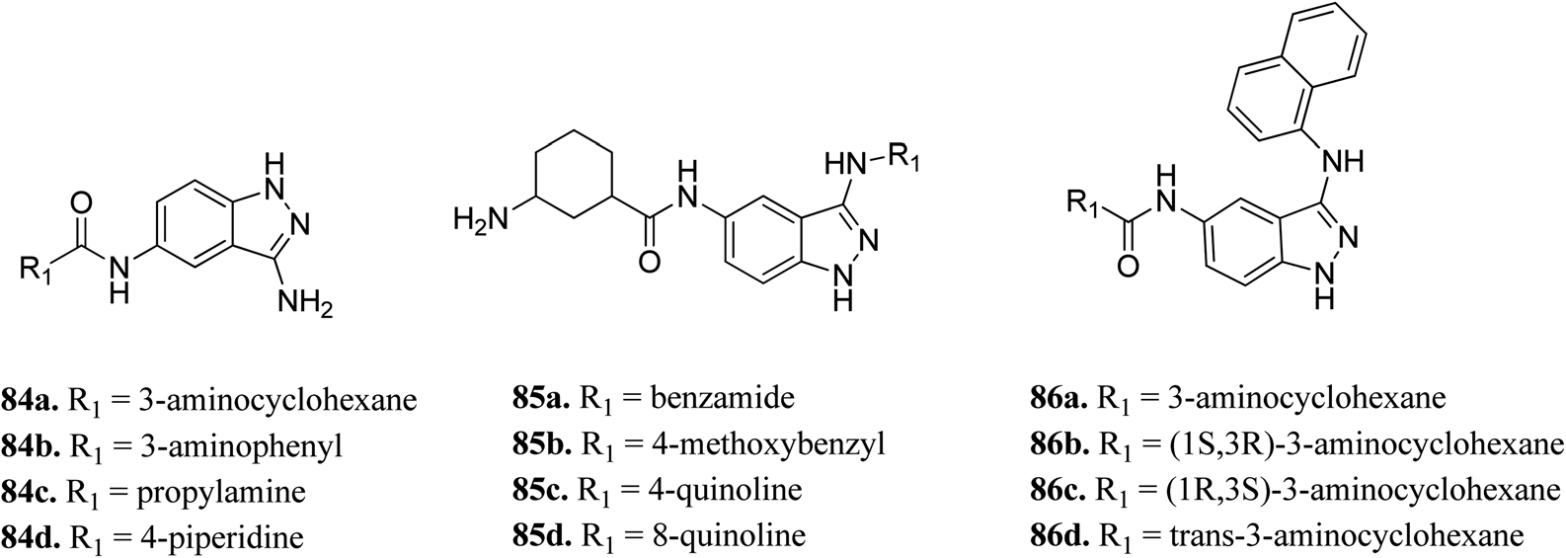
*In silico* HTS and structure-based optimization of indazole derivatives as ULK-1 inhibitors.

Wood *et al.* reported the *in silico* HTS and structure-based optimization of indazole derivatives (Fig. 38) as ULK-1 inhibitors.^152^ Initial screening led to the discovery of SR-17398 with ULK-1 with the IC_50_ value of 22.4 μM. Substitution by an amino group at the 3-position of the indazole ring afforded 84a with ULK-1, IC_50_ = 368 nM. Further, replacement of the cyclohexyl group with a 3-aminohexyl (84b) or 3-aminopropyl group (84c) led to a decrease in activity (ULK-1, IC_50_ = 18.1 μM and >33 μM), while piperidine substitution gave reasonable activity (84d, ULK-1, IC_50_ = 560 nM). Substitution with a naphthyl ring resulted in a significant enhancement in activity (ULK-1, IC_50_ = 11 nM), whereas the quinoline derivatives (85c and 85d) possessed lower activity than 86a. The *cis*-isomers (86b and 86c, ULK-1, IC_50_ = 3 and 45 μM, respectively) were found to be less active than the *trans*-isomer (86d, ULK-1, IC_50_ = 24 nM).

### (xviii) P21-activated kinase (PAK1) inhibitors

P21-activated kinases (PAKs) belong to the non-receptor serine/threonine protein kinase family, which play an important role in many signaling pathways, and thus contribute to cancer development.^153^ They can be classified into two groups, where group-I includes PAK1-3 and group-II includes PAK4-6.^154^ Abnormal expression of PAK1 is associated with skin cancer,^155–157^ breast cancer^158^ and pancreatic cancer.^159^

**Fig. 39:**
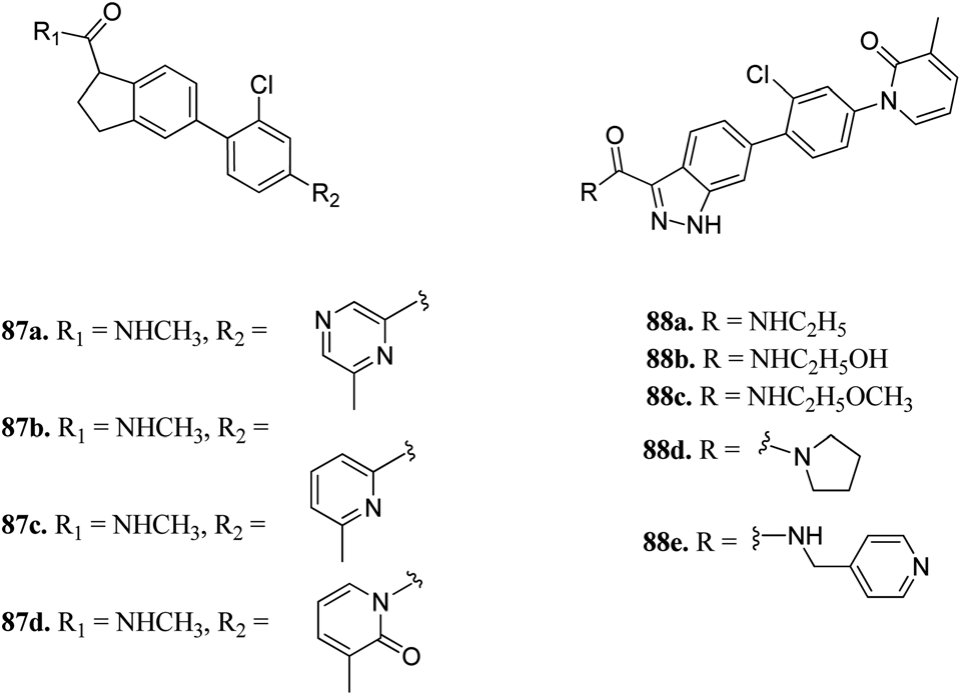
1*H*-Indazole-3-carboxamide derivatives as inhibitors of PAK1.

Zhang *et al.* reported 1*H*-indazole-3-carboxamide derivatives (Fig. 39) as inhibitors of PAK1.^160^ Initial screening of 235 molecules resulted in compound 87a (PAK1, IC_50_ = 5 μM), which was further utilized for scaffold modification. Introducing a hydrophobic moiety onto the chlorophenyl group led to derivatives with enhanced activity against PAK1 (87b; IC_50_ = 159 nM, 87c; IC_50_ = 52 nM and 87d; IC_50_ = 16 nM). Interestingly, compound 88f was found to be 1000-fold more active against PAK1 compared to PAK4 and was selective in the presence of other 28 kinases. It also displayed low hERG channel activity and was found to decrease the invasion of HCT116 and MDA-MB-231 cancer cells.

### (xix) Leucine-rich repeat kinase 2 (LRRK2) inhibitors

Leucine-rich repeat kinase 2 (LRRKs2) belongs to the leucine-rich repeat kinase family and overexpression of LLRK2 is related to the inhibition of proliferation and migration of cellular events related to thyroid cancer.^161,162^

**Fig. 40:**
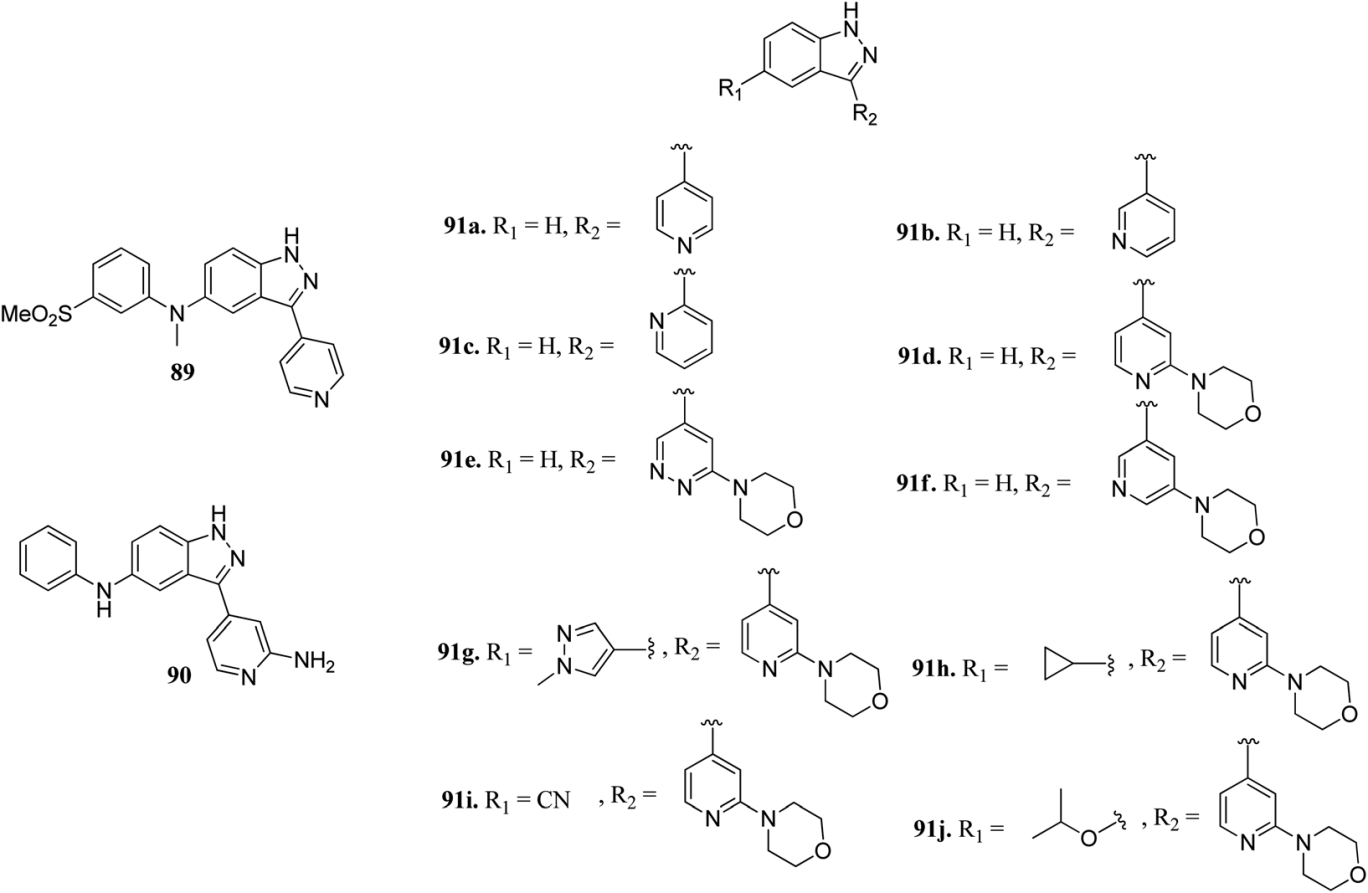
Truncated indazole derivatives as leucine-rich repeat kinase 2 (LRRK2) inhibitors.

Scott *et al.* reported truncated indazole derivatives (Fig. 40) as leucine-rich repeat kinase 2 (LRRK2) inhibitors and compared their activity with reference to compounds 89 and 90 (IC_50_ = 44 and 728 nM, respectively), which were the results of initial high throughput screening of a library of compounds.^163^ Thus, the 3- and 4-pyridyl analogs (91a and 91b; IC_50_ = 904 and 1231 nM, respectively) were found to be more potent than the 2-pyridyl analog (91c; IC_50_ = 6186 nM). Further, substitution of the pyridyl ring with the piperazine moiety significantly enhanced the activity (91d; IC_50_ = 12 nM), which was 21-fold more than regioisomer 91e (IC_50_ = 256 nM) and 11-fold more than 91f (IC_50_ = 134 nM). Interestingly, the introduction of a pyrazole group at C5 led to a series of highly potent compound 91g with IC_50_ < 0.6 nM. Compound 91h with an isopropoxy substituent possessed high activity (IC_50_ = 7 nM) with excellent selectivity (17 out of 306 kinases studied).

### (xx) Phosphatidylinositol-3-kinase (PI3K) inhibitors

Phosphatidylinositol-3-kinases are a family of enzymes that are responsible for the regulation of cell growth, angiogenesis, metabolism, motility and survival.^164,165^ Dysregulation of the PI3K pathway leads to colorectal and breast cancer and hematologic malignancies.^166^ Therefore, inhibition of PI3K is associated with the suppression of cellular proliferation and enhancement of cellular death.^167^

**Fig. 41:**
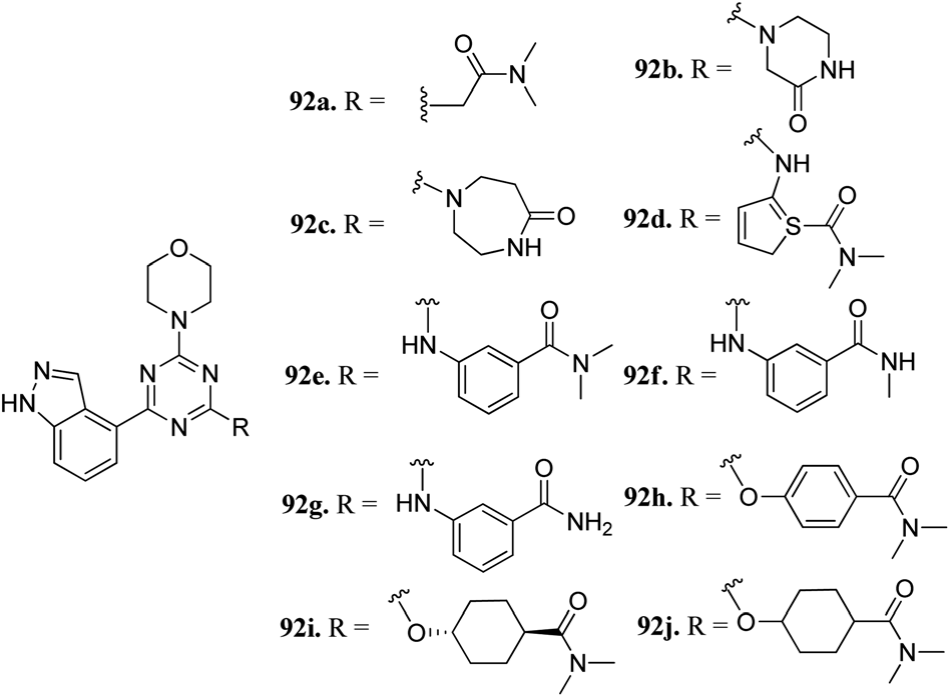
Indazole-substituted morpholino-triazines as PI3 kinase inhibitors.

Dugar *et al.* reported indazole-substituted morpholino-triazines (Fig. 41) as PI3 kinase inhibitors.^168^ The lead compound 92a (IC_50_ = 1.10 μM) was used as a starting point for further optimization. Replacement of the glycine amide chain with a conformational restricted 6- or 7-membered ring led to an improvement in cellular and antiproliferation activity (92b; PI3, IC_50_ = 0.40 μM; A2780, IC_50_ = 3.97 μM and 92c, PI3, IC_50_ = 0.20 μM; and A2780, IC_50_ = 2.64 μM). Further optimization of the spacer and orientation of the terminal amide led to compounds 92d–g with enhanced activity. Interestingly compound 92h with *para*-substitution of the amide group exhibited remarkable activity (PI3, IC_50_ = 0.06 μM and A2780, IC_50_ = 0.52 μM). Further, compounds 92h–j did not show any inhibition of CYP3A4, CYP2C19 and CYP2D6 and were not predicted to have any hERG liability.

### (xxi) Tyrosine threonine kinase (TTK) inhibitors

Tyrosine threonine kinase (TTK) is a dual-specific kinase with upregulation found in different type of cancers such as breast cancer, anaplastic thyroid carcinoma (ATC), pancreatic ductal adenocarcinoma (PDAC) and human hepatocellular carcinoma (HCC), and therefore, inhibition of TTK results in the inhibition of cell growth and enhancement of cell apoptosis.^169–172^

**Fig. 42:**
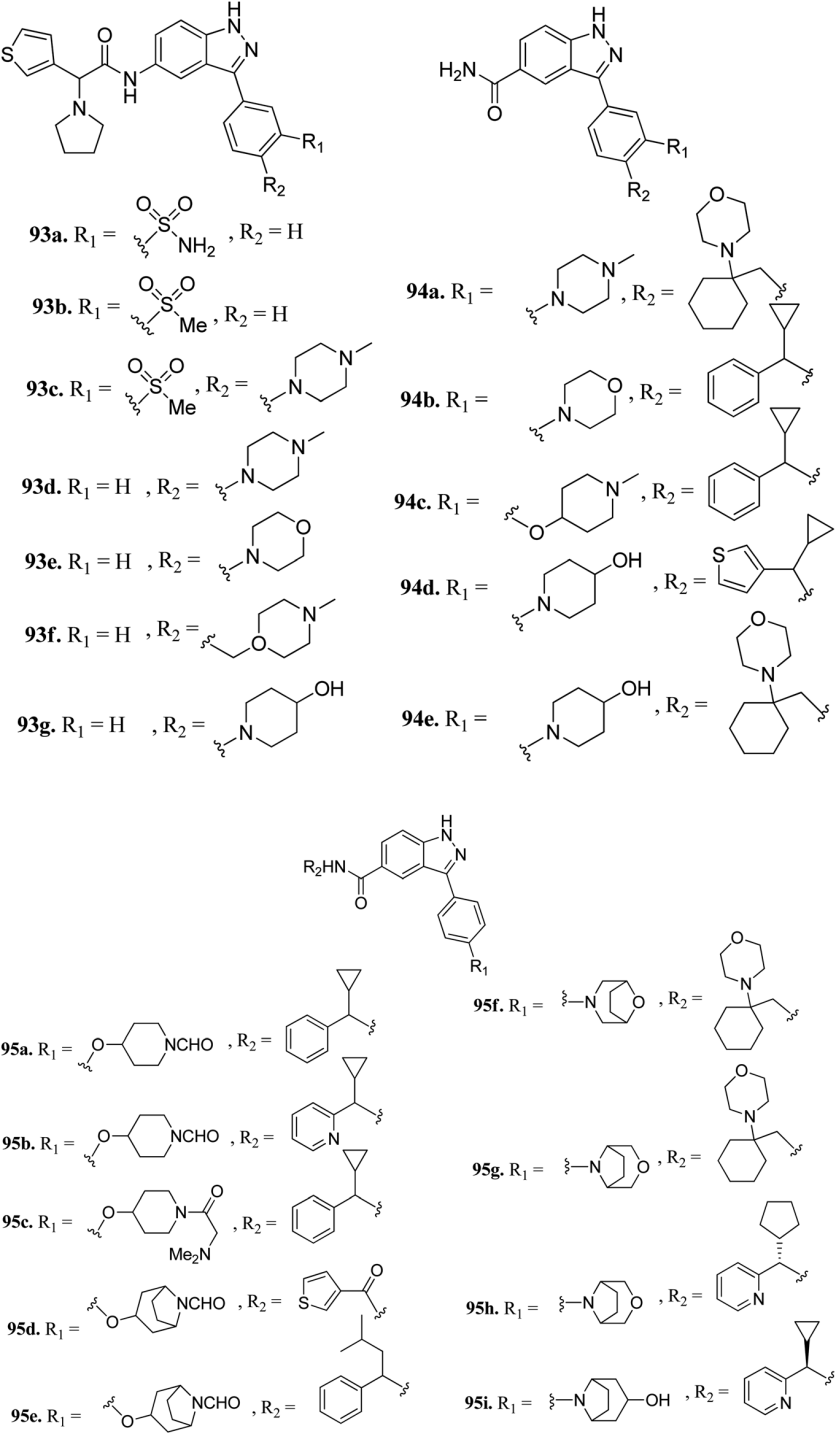
3-(4-(Heterocyclic)phenyl)-1*H*-indazole-5-carboxamide derivatives as tyrosine threonine kinase (TTK) inhibitors.

Liu *et al.* reported 3-(4-(heterocyclic)phenyl)-1*H*-indazole-5-carboxamide derivatives (Fig. 42) as tyrosine threonine kinase (TTK) inhibitors.^173^ Modification of sulfonamide (93a, IC_50_ = 0.0029 μM) to a sulfone derivative (93b, IC_50_ = 0.0059 μM) was well tolerated, but a reduction in the polar surface area (93c, IC_50_ = 0.018 μM) led to a 4-fold decrease in potency. Also, 4-monosubstituted derivatives (93d–g) exhibited acceptable TTK activity (IC_50_ = 0.003–0.007 μM) together with good cell viability (GI_50_ < 0.057 μM). Further optimization led to carboxamide analogs with different heterocyclic moieties (94a and b, and 94c–e) with TTK IC_50_ in the nano molar range. Further, the bridged formylated derivatives 95d–g retained potency, among which 95g (TTK, IC_50_ = 0.0011 μM and AURKB/INCENP, IC_50_ = 0.80 μM) retained potency and selectivity against AURKB/INCENP. Further optimization led to the discovery of 95h, showing good activity (TTK, IC_50_ = 0.0012 μM; HCT116 and GI_50_ = 0.003 μM) and high selectivity over AURKB/INCENP. Further studies suggested that compound 95i has potency of *K*_i_ = 0.7 nM and was found to be selective against a panel of 278 human kinases including the mutant form of cKit, Ret, c-Jun N-terminal kinase 3 (JNK3), PLK4 and mitogen-activated protein kinase 2 (MAPK2) compared to sunitinib.

## 3. Multiple kinase inhibitors

### (i) Fibroblast growth factor receptor-1 (FGFR1) and histone deacetylase (HDAC) inhibitors

**Fig. 43:**
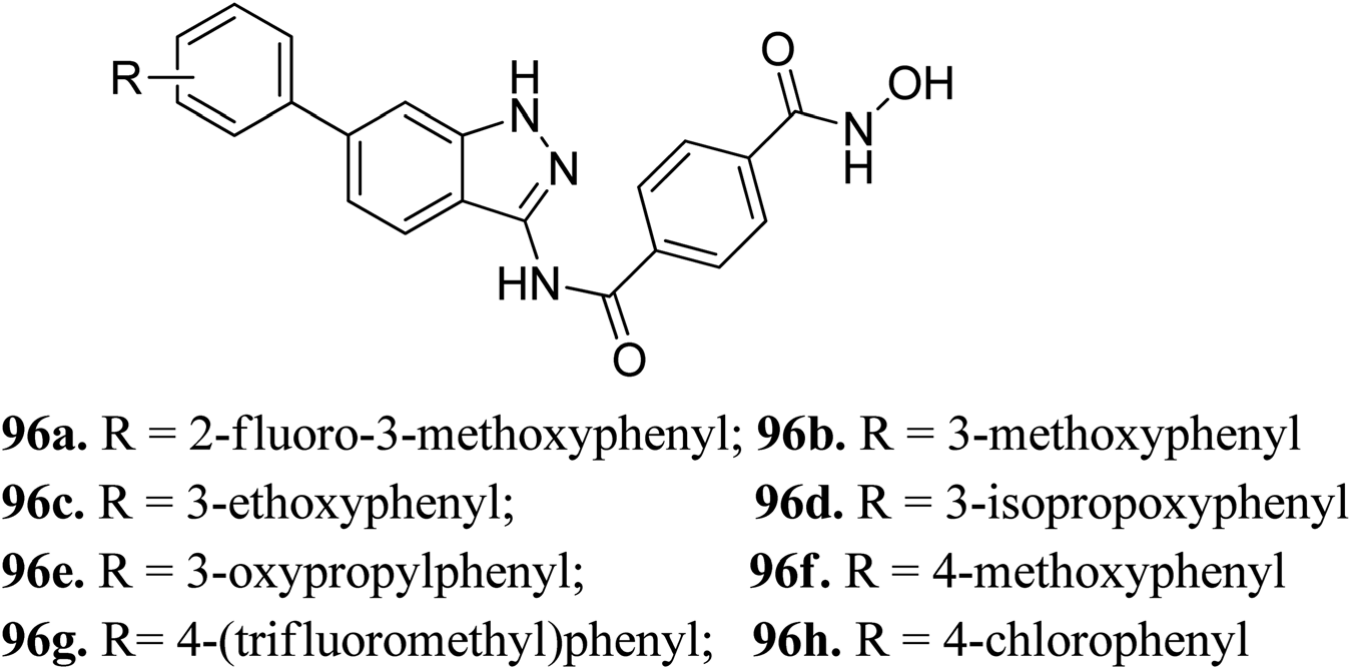
Indazole derivatives as dual inhibitors of FGFR1 and HADC.

Liu *et al.* reported a series of indazole derivatives (Fig. 43) as dual inhibitors of FGFR1 and HDAC.^174^ Their studies suggested that the removal of a fluorine atom of 96a (IC_50_ = 58 nM) led to a decrease in HDAC6 inhibition with IC_50_ = 132 nM of the resulting compound 96b. Conversely, the presence of a 3-ethoxy group (96c; IC_50_ = 34 nM) showed better activity than the compounds possessing methoxy (96b; IC_50_ = 132 nM), isopropoxy (96d; IC_50_ = 78 nM) and propoxy groups (96e; IC_50_ = 132 nM). In addition, 96f also showed better cellular inhibition against MCF-7 with IC_50_ = 9 μM. The presence of an electron donor group at the *para*-position of the phenyl ring led to enhanced activity, as found in the case of 96f (IC_50_ = 41 nM) compared to 96g (IC_50_ = 103 nM) and 96h (IC_50_ = 281 nM). Moreover, the selectivity of these synthesized compounds was found to be significantly higher for HDAC6 compared to HDAC1 and HDAC8. Compound 96c turned out to be the most effective with the highest HDAC6 activity but moderate FGER1 activity.

**Fig. 44:**
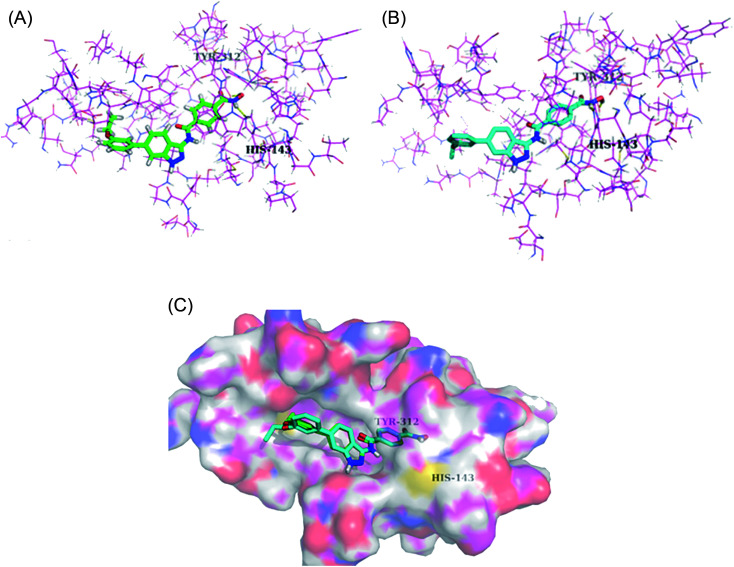
(a) Docking mode of compound 96c with HDAC6 (reproduced with permission; License Number: 5090111285759). (b) Docking mode of compound 96e with HDAC6 (reproduced with permission; License Number: 5090111285759). (c) Superposed docking poses of 96c (green) and 96e (blue) with HDAC6 (reproduced with permission; License Number: 5090111285759).

Derivatives 96c and 96e were further studied for their docking with HDAC6 (Fig. 44). It was observed that the hydroxamic acid group of both derivatives chelated with zinc ions to enter the active site. Further, indazole group formed hydrophobic interactions with HDAC6, whereas the cap groups occupied the space outside the groove. The oxygen atom of the hydroxamic acid group formed a hydrogen bond with Tyr312 and the nitrogen atom formed a hydrogen bond with His143.

### (ii) Epidermal growth factor/human epidermal growth factor receptor-2 (EGFR/HER-2) inhibitors

**Fig. 45:**
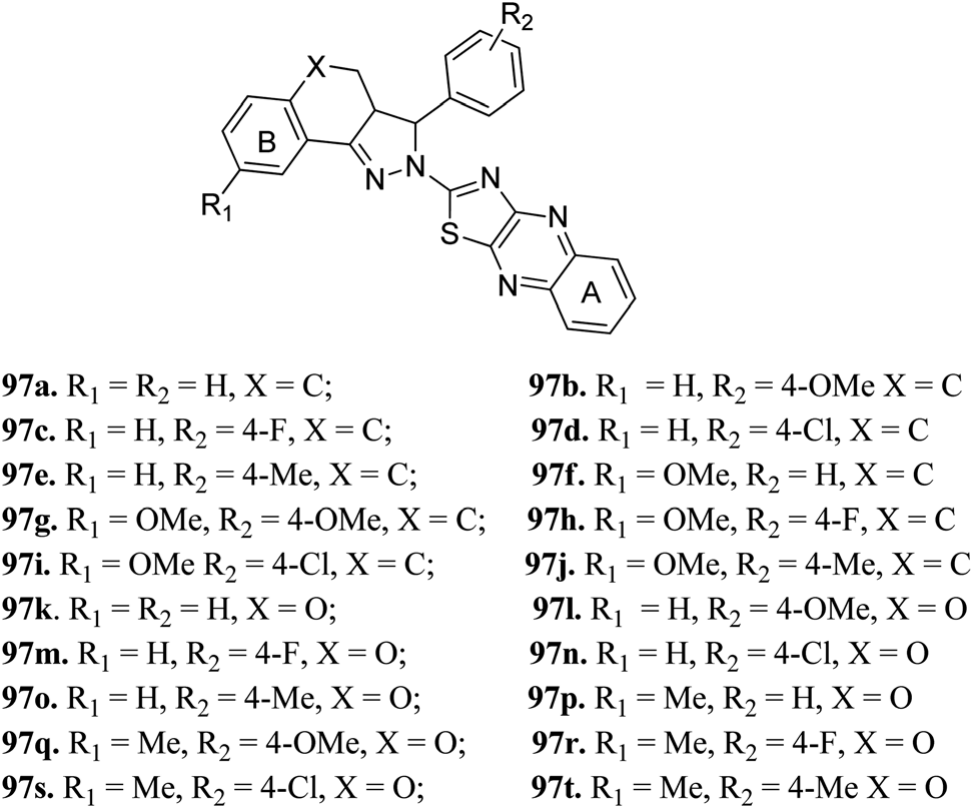
3,3*a*,4,5-Tetrahydro-2*H*-benzo[*g*] indazole derivatives of quinoxaline as EGFR/HER-2 dual inhibitors.

Zong *et al.* reported 3,3*a*,4,5-tetrahydro-2*H*-benzo[*g*] indazole derivatives (Fig. 45) of quinoxaline as EGFR/HER-2 dual inhibitors.^175^ The structure–activity relationship (SAR) studies suggested that the presence of electron-donating groups on ring A led to higher enzymatic activity than the presence of electron-withdrawing groups (97b > 97e > 97a > 97d > 97c with EGFR, IC_50_ = 2.26, 8.26, 9.82, 15.67, 21.24 μM and HER-2, IC_50_ = 3.68, 12.38, 11.43, 26.58 and 18.43, respectively). Also, the derivatives with a methoxy group at 4-position of ring B (97f–j) were more active than those with a methyl in the hydrogen substituents (97k–t). Interestingly, 97g was found to be the most active among the series with IC_50_ = 0.28 and 1.26 μM for EFGR and HER-2, respectively.

**Fig. 46:**
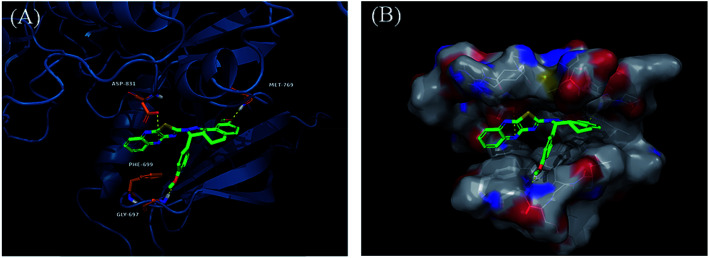
(A) Predicted binding model of 97g with EGFR (reproduced with permission). (B) Surface of the binding site of 97g with EGFR (reproduced with permission).

**Fig. 47:**
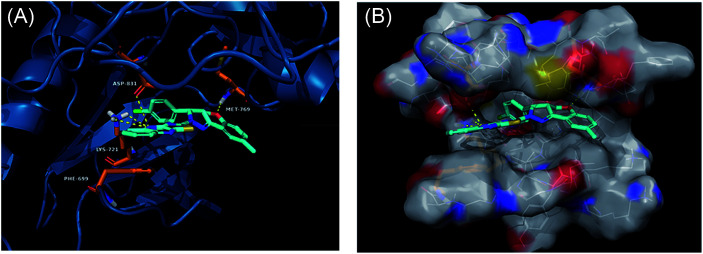
(A) Predicted binding model of 97r with EGFR (reproduced with permission). (B) Surface of the binding site of 97r with EGFR (reproduced with permission).

Derivatives 97g and 97r were further studied for their docking with EGFR (Fig. 46 and 47), which suggested the hydrogen bonding of the methoxy oxygen of 97g with Met769 and Gly697 with additional hydrogen bonding between the nitrogen of the quinoxaline group with Asp831. Further, the terminal phenyl moiety of 97g participated in π–π interaction with Phe699. This was also observed in the docking study of 97r. Also, the oxygen of the tricyclic ring of 97r was observed to form a hydrogen bond with Met769, whereas the nitrogen of the quinoxaline group participated in hydrogen bonding with Asp831 and lys721.

### (iii) Serine/threonine-protein kinase 1 (SGK1), tyrosine-protein kinase receptor (Tie2) and proto-oncogene tyrosine-protein kinase (SRC) inhibitors

**Fig. 48:**
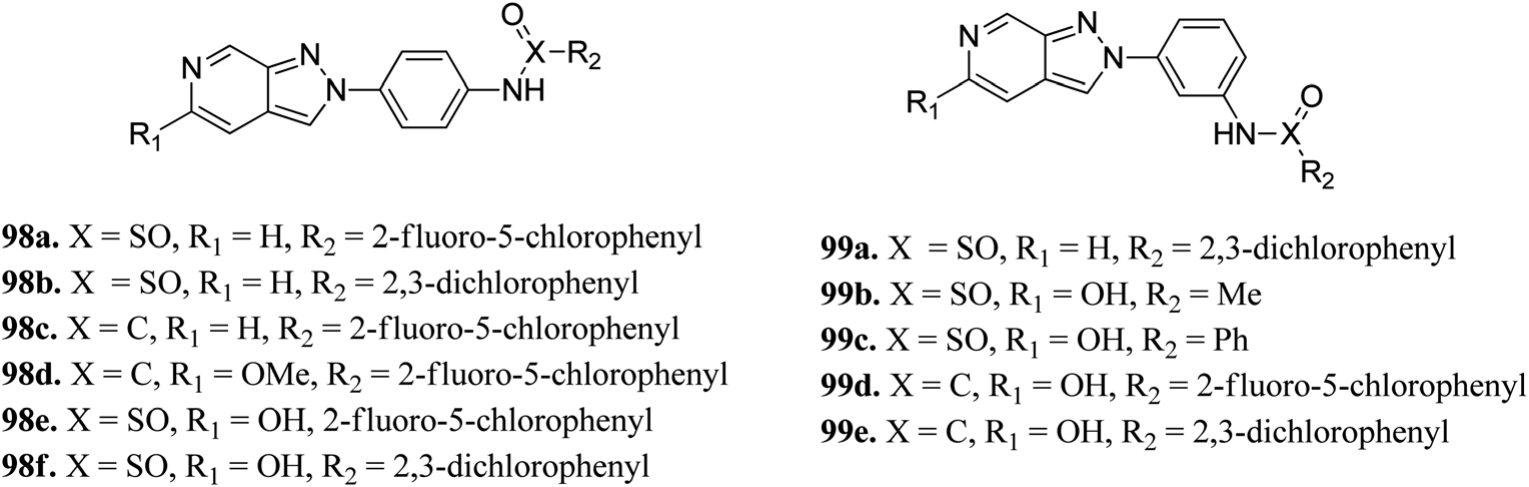
2*H*-Indazoles derivatives as potential inhibitors of SGK1, Tie2 and SRC.

Schoene *et al.* reported the synthesis of 2*H*-indazole derivatives (Fig. 48) as potential inhibitors of SGK1, Tie2 and SRC.^176^ All the synthesized compounds showed at least 70% inhibition against at least one of the three kinases with almost equivalent activity against SGK1 and Tie2 (98a–d, 98f and 99a and b). 2-Fluoro-3-chloro-substitution and 2,3-dichloro derivatives were found to be superior to the unsubstituted phenyl derivatives. Interestingly, only the 5-hydroxy-6-aza-2*H*-indazole motif (99) showed significant SRC inhibition. Further, docking studies of 99e with SGK1 (PDB ID 3HDM) showed high affinity (2.13–221.15 μM), which is in line with its IC_50_ value of 733 nM.

**Fig. 49:**
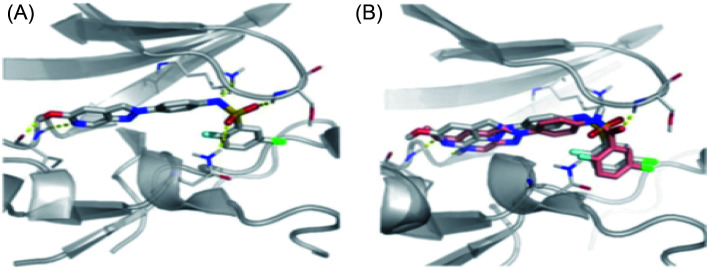
(a) Docking structure of 98e with SGK1 structure PDB ID: 3HDM. (b) Docking structure of 98e (grey) and 98d (salmon) with SGK1 structure PDB ID: 3HDM.

The docking studies of 98e with SGK1 PDB ID 3HDM suggested that the azaindazole head forms hydrogen bonds with Ile179 and/or Asp177. In addition, sulfonamide formed a hydrogen bond with Asn277 or Ser108. Conversely, the methyl group of 98d was exposed to the solvent front, which forced the orientation of 98d into the front pocket than 98e. In addition, the carboxylic group formed hydrogen bonding interactions with Ser108, and the nitrogen atom formed hydrogen bonding with Lys127 (Fig. 49).

### (iv) Tie2, vascular endothelial growth factor-2 (VEGFR-2) and erythropoietin-producing human hepatocellular-B4 (EphB4) inhibitors

**Fig. 50:**
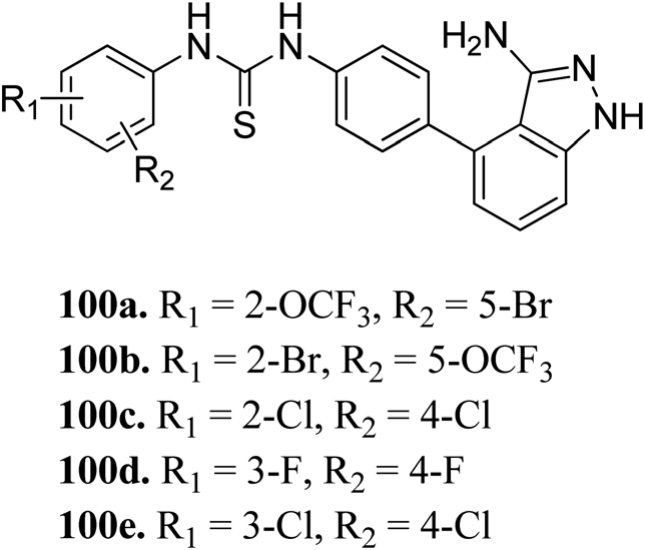
Diaryl thiourea derivatives of 1*H*-indazole-3-amine as multiple kinase inhibitors.

Sun *et al.* reported the design and synthesis of diaryl thiourea derivatives (Fig. 50) of 1*H*-indazole-3-amine as multiple kinase inhibitors.^177^ Their studies suggested that these derivatives (100a–e) exhibit simultaneous activity against Tie2, VEGFR-2 and EphB4 with IC_50_ of less than 50 nM in all cases. Compound 100a emerged as the most potent derivative with IC_50_ values of 3.45, 2.13 and 4.71 nM against VEGFR-2, Tie2 and EphB4, respectively, supporting the importance of the 1*H*-indazol-3-amine moiety, which acts as a hinge binding group between the kinases under study, as confirmed by the docking studies. In accordance with its kinase inhibition, 100a was found to inhibit the growth of 9 cancer cell lines (human liver cancer cell line; HepG2, SMMC-7721, human pancreatic cancer cell line; MIAPaCa-2, A431, human gastric cancer cell lines; MGC-803 and MKN28, human thyroid-carcinoma cancer cell-lines; 8505C, K1 and BCPAP) with IC_50_ values between 1.80–13.26 μM.

**Fig. 51:**
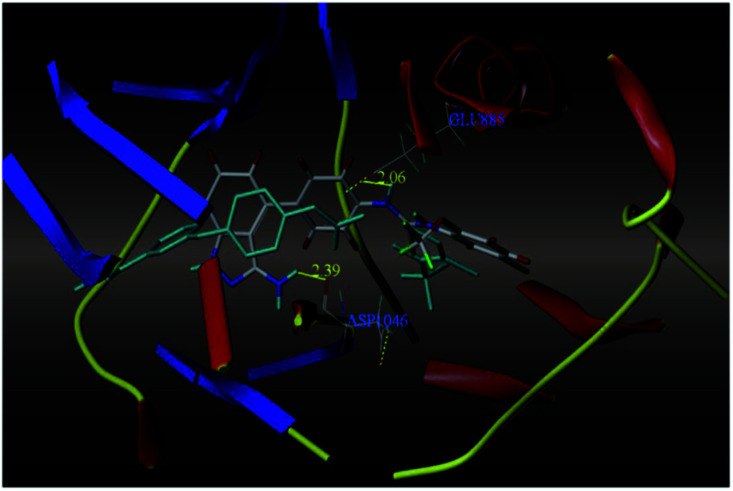
Docking structure of 100a with VEGFR-2 (reproduced with permission; License Number: 5087591358694).

**Fig. 52:**
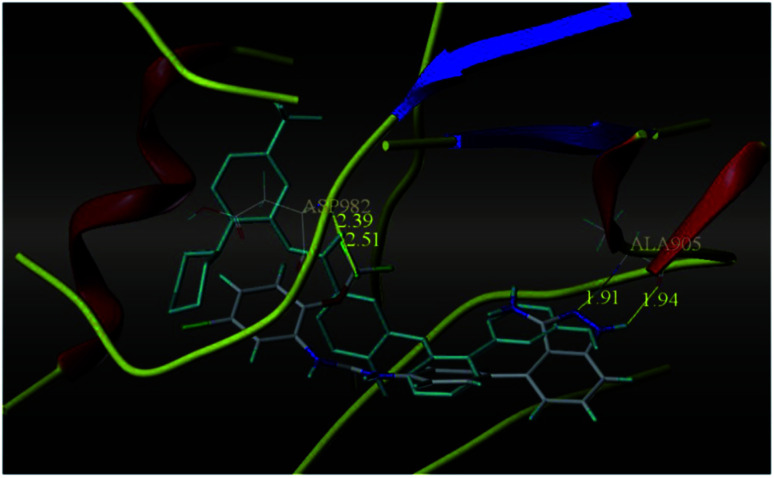
Docking structure of 100a with Tie-2 (reproduced with permission; License Number: 5087591358694).

**Fig. 53:**
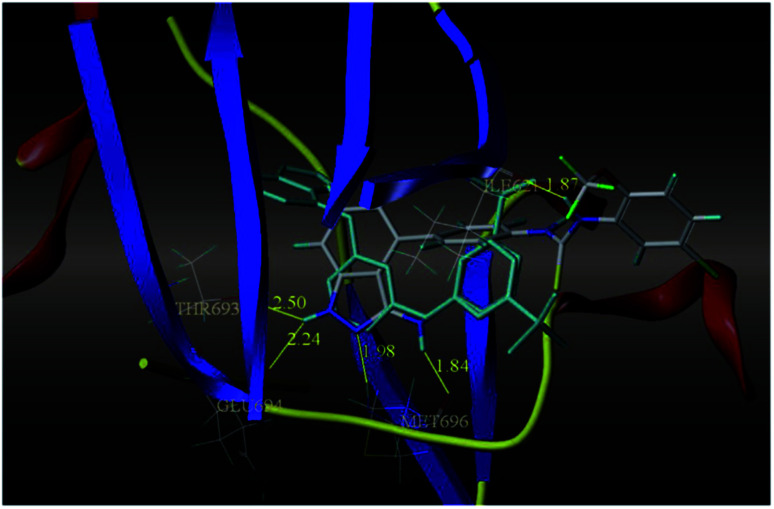
Docking structure of 100a with EphB4 (reproduced with permission; License Number: 5087591358694).

The most potent derivative 100a was subjected to docking studies with the ATP pocket of VEGFR-2 (Fig. 51), Tie-2 (Fig. 52) and EphB4 (Fig. 53). Studies suggested that the amine group of the 1*H*-indazol-3-amine of 100a participated in hydrogen bond formation with Asp1046 of VEGFR-2 and with Glu885 of Tie-2. In addition, the 1*H*-indazol-3-amine group formed two additional hydrogen bonds with Ala 905 of Tie-2. The 1*H*-indazol-3-amine group also formed hydrogen bonds with Met 696, Thr 693 and Glu 694 of EphB4 with additional hydrogen bond formation between the NH of urea and CO of Ile 621.

## 4. Multi target inhibitors

**Fig. 54:**
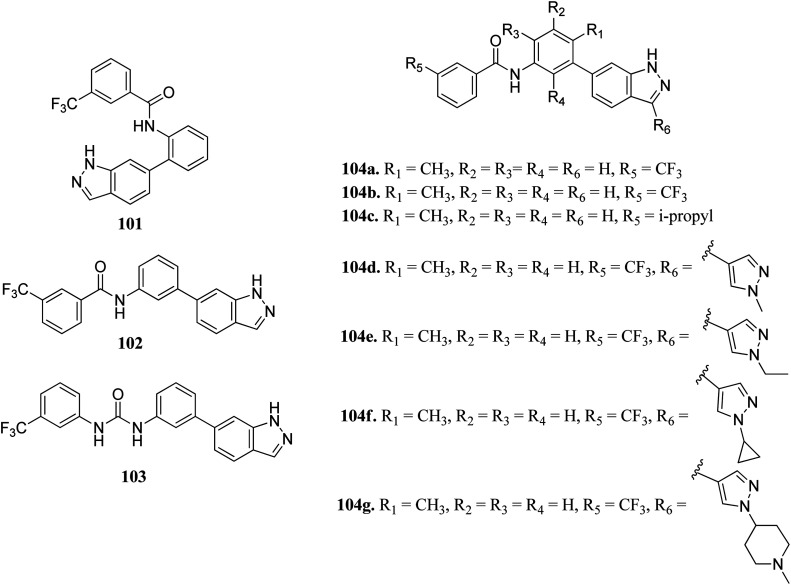
3-Substituted indazole derivatives as multi-target kinase inhibitors.

Wang *et al.* reported a series of 3-substituted indazole derivatives (Fig. 54) as potential multi-target kinase inhibitors.^178^ Shifting of the *N*-(3-substituted)-3-(trifluoromethyl) benzamide moiety from the 6-position (101; 14% inhibitory rate) to the 5-position (102; 46.8% inhibitory rate) resulted in a significant decrease in activity in DDR2 according to the enzyme assay studies. Further optimization led to the formation of compound 104a with IC_50_ = 5.5 nM. Reversing the amide substituent of 104a and then with isopropyl group led to a further improvement of activity to IC_50_ = 1.5 nM and 1.2 nM for compounds 104b and 104c, respectively. Further optimization led to the formation of 104d, 104e, 104f and 104g, which possessed promising activity against FGFR1 and DDR2. Further, 104f was also found to active against KG1, NCI-H716, SNU-16 and a human transitional cell carcinoma cancer cell line (UMUC14) with IC_50_ = 108.4, 31.8, 93.4 and 306.6 nM, respectively. Further drug-like studies supported that 104d can be a potential drug for kinase inhibition.

**Fig. 55:**
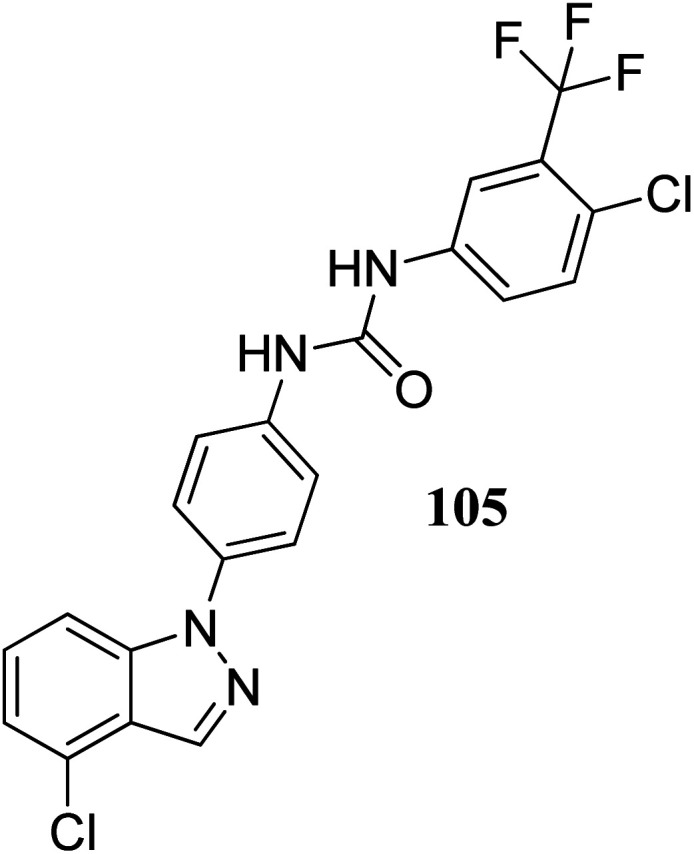
Diarylurea derivatives as multi-kinases inhibitor.

Lu *et al.* discovered diarylurea derivatives (Fig. 55) as multi-kinase inhibitors.^179^ The KINOMEscan™ system was used to study the binding assay of HL-40 against 450 human kinases at 10 μM concentration, which suggested FLT3, CDK11, HIPK4, CDK8, DDR1, PDGFRb and KIT targeting compound 105. Compound 105 was found to bind selectively to tyrosine-protein kinase (c-Kit), platelet-derived growth factor receptor beta (PDGFRb) and FLT3 with *K*_d_ values of 68.5, 140 and 375 nM, respectively. The pharmacokinetic studies further suggested that compound 105 did not absorb rapidly in rat plasma given that the time to reach the maximum plasma concentration was 5.5 h. Further, compound 105 exhibited strong activity against an acute promyelocytic leukemia-derived cancer cell line (NB-4), human melanoma cancer cell line (SK-MEL-31 and SK-MEL-3) with IC_50_ values of 1.34, 1.77 and 1.12 μM, respectively.

## 5. Conclusion and future perspectives

In this review, we discussed and highlighted the importance of indazole derivatives as kinase inhibitors. The easy synthesis of the derivatives of this core offers the scope of structure–activity studies with electron-donating and electron-withdrawing substituents around this versatile pharmacophore and their application in drug development and medicinal chemistry. The biological activities of indazole were demonstrated as kinase inhibitors including, tyrosine kinase, cyclin-dependent kinase, aurora kinase, EGFR, and VEGFR and their application against many cancer diseases such as breast cancer, non-small cell lung cancer, colon cancer, stomach cancer and liver cancers. Although multiple hypotheses have been proposed for the applications of indazole derivatives for kinase inhibitory activity, mono-kinase inhibition is the most widely accepted and considered to be a convincing target for the development of new indazole-based drug candidate. Some drugs with an indazole core are already in the market such as axitinib, linifanib, niraparib, and pazopanib, which show mono-kinase inhibitory activity. Thus, the exploration of the indazole scaffold will continue to identify many more biologically active kinase inhibitors in the future. The *in vitro* and *in vivo* activities of indazole derivatives are useful for future innovation. Progress in the development of indazole-based new targets for preventing the uncontrolled division of cells has been encouraging, but several drug candidates have unfortunately failed at various stages of clinical trials. These failures may be due to the interference by the drug candidates in many disease pathways, and thus the success rate to a particular target is very low. Current approaches to targeting kinase enzymes have shown adverse side effects, which are attributed to their vital role in many other biological processes. In fact, the actual cause and realistic molecular targets need to be discovered and validated based on existing and new knowledge. To increase the efficiency, selective targeting should be actively pursued to reduce side effects, which is the main aim of cancer drugs. The indazole moiety has become a new drug candidate for anticancer activity, and the synthetic and clinical research communities need to collaborate to provide better and faster solutions in terms of dealing with new target for the effective control of cancer cells. Although active research groups are involved in developing indazole-based targets, there is no dedicated grand initiative in the area of clinical trials, which is responsible for the lag in activity based in this moiety. Further, we are hopeful that research groups will give their full efforts, and knowledge accumulated over the years will supplement the ongoing and forthcoming efforts in drug discovery to successfully develop new targets for the effective diagnosis of cancer in the near future. This article provides comprehensive and target-oriented information on the indazole core to synthetic and medicinal chemists for the development of potent and novel indazole derivatives as kinase inhibitors and new cancer therapy in the future.

## Conflicts of interest

The author declares no conflict of interest.

## Supplementary Material
